# Prevalence, awareness, treatment, and control of hypertension in China, 2004-18: findings from six rounds of a national survey

**DOI:** 10.1136/bmj-2022-071952

**Published:** 2023-01-11

**Authors:** Mei Zhang, Yu Shi, Bin Zhou, Zhengjing Huang, Zhenping Zhao, Chun Li, Xiao Zhang, Guiyuan Han, Ke Peng, Xinhua Li, Youfa Wang, Majid Ezzati, Limin Wang, Yichong Li

**Affiliations:** 1 National Center for Chronic and Noncommunicable Disease Control and Prevention, Chinese Center for Disease Control and Prevention, Beijing 100050, China; 2 National Clinical Research Center for Cardiovascular Diseases, Heart Failure Ward, Fuwai Hospital Chinese Academy of Medical Sciences, Shenzhen, Guangdong Province, China; 3 Department of Epidemiology and Biostatistics, School of Public Health, Imperial College London, London, UK; 4 MRC Centre for Environment and Health, School of Public Health, Imperial College London, London, UK; 5 The Abdul Latif Jameel Institute for Disease and Emergency Analytics, School of Public Health, Imperial College London, London, UK; 6 People’s Medical Publishing House, Beijing, China; 7 Global Health Institute, School of Public Health, Xi’an Jiaotong University, Xi’an, China

## Abstract

**Objective:**

To assess the recent trends in prevalence and management of hypertension in China, nationally and by population subgroups.

**Design:**

Six rounds of a national survey, China.

**Setting:**

China Chronic Disease and Risk Factors Surveillance, 2004-18.

**Participants:**

642 523 community dwelling adults aged 18-69 years (30 501 in 2004, 47 353 in 2007, 90 491 in 2010, 156 836 in 2013, 162 293 in 2015, and 155 049 in 2018).

**Main outcome measures:**

Hypertension was defined as a blood pressure of ≥140/90 mm Hg or taking antihypertensive drugs. The main outcome measures were hypertension prevalence and proportion of people with hypertension who were aware of their hypertension, who were treated for hypertension, and whose blood pressure was controlled below 140/90 mm Hg.

**Results:**

The standardised prevalence of hypertension in adults aged 18-69 years in China increased from 20.8% (95% confidence interval 19.0% to 22.5%) in 2004 to 29.6% (27.8% to 31.3%) in 2010, then decreased to 24.7% (23.2% to 26.1%) in 2018. During 2010-18, the absolute annual decline in prevalence of hypertension among women was more than twice that among men (−0.83 percentage points (95% confidence interval −1.13 to −0.52) *v* −0.40 percentage points (−0.73 to −0.07)). Despite modest improvements in the awareness, treatment, and control of hypertension since 2004, rates remained low in 2018, at 38.3% (36.3% to 40.4%), 34.6% (32.6% to 36.7%), and 12.0% (10.6% to 13.4%). Of 274 million (95% confidence interval 238 to 311 million) adults aged 18-69 years with hypertension in 2018, control was inadequate in an estimated 240 million (215 to 264 million). Across all surveys, women with low educational attainment had higher prevalence of hypertension than those with higher education, but the finding was mixed for men. The gap in hypertension control between urban and rural areas persisted, despite larger improvements in diagnosis and control in rural than in urban areas.

**Conclusions:**

The prevalence of hypertension in China has slightly declined since 2010, but treatment and control remain low. The findings highlight the need for improving detection and treatment of hypertension through the strengthening of primary care in China, especially in rural areas.

## Introduction

Hypertension is a leading risk factor for stroke, ischaemic heart disease, other cardiovascular diseases, and chronic kidney disease and is responsible for more than 10 million deaths worldwide each year.^1^ In China in 2019, more than 25 million disability adjusted life years for stroke were attributed to high systolic blood pressure,^2^ and stroke is the leading cause of mortality.^3^ Overwhelming evidence indicates that control of hypertension is associated with important reductions in cardiovascular events and deaths.^4^

In recent years the prevalence of hypertension has declined and its treatment and control have improved substantially in some high income countries, such as Canada, South Korea, Germany, and Chile.^5^ These countries usually have well constructed primary care systems, high insurance coverage, widely adopted evidence based hypertension guidelines, and, in some countries, nationwide health check-ups and screening.^5^ ^6^ In the late 2000s, China started multiple nationwide programmes with packages for the prevention and control of hypertension, including the China Healthy Lifestyle for All Initiative in 2007,^7^ National Basic Public Health Services Program in 2009,^8^ and National Demonstration Areas for Comprehensive Prevention and Control of Non-communicable Diseases in 2011.^9^ To evaluate the impact of these national public health programmes and guide future improvements, assessment of recent trends in the prevalence of hypertension and its management and control in China is essential after the implementation of these initiatives.

Several nationally representative studies have reported the prevalence, awareness, treatment, and control of hypertension in China since the 2000s, most of which were performed before 2010.^10^ ^11^ ^12^ ^13^ These studies adopted different sampling schemes and survey methods, with key differences in age ranges, procedures, and definitions of hypertension, making it difficult to assess trends. The lack of reliable, consistent data on recent trends in the prevalence, management, and control of hypertension hampers the essential understanding of the scale of, and change in, the burden of hypertension in China, and where intervention is needed the most.

Based on data from six rounds of a nationally representative survey with broadly consistent design and protocol, we examined trends in the prevalence of and number of adults with hypertension in China during 2004-18, and the extent of awareness, treatment, and control of hypertension and how each varied by sex, age, geography, and socioeconomic status.

## Methods

### Data sources

We used data from the China Chronic Disease and Risk Factor Surveillance (CCDRFS) survey. The history, development, and design of this survey have been described previously.^14^ Briefly, the survey was established in 2004 to provide periodic nationwide information on the prevalence and distribution of major non-communicable diseases and associated behavioural and metabolic risk factors in the Chinese population. To date, six rounds of the survey have been conducted: in 2004, 2007, 2010, 2013, 2015, and 2018. Each roundadopted a similar sampling scheme with a stratified, clustered, and multistage design to select a random sample from the Chinese adult population. In 2004, the survey included the 79 districts and counties in the national Disease Surveillance Points system, which were randomly selected from all 31 provinces, autonomous regions, and municipalities of mainland China.^15^ The number of surveillance districts and counties was expanded to 161 in 2007 and 2010. Since 2013, 298 districts and counties have been enrolled into the China Chronic Disease and Risk Factor Surveillance, making the survey sample provincially representative. Supplement 1 and a previous report provide details of population coverage, sample design, and weighting.^16^

Across all six rounds of the survey, 776 571 individuals were invited and 746 020 participated (response rate 96.1%), including 33 051 (99.6%) in 2004, 51 050 (99.1%) in 2007, 98 174 (90.5%) in 2010, 189 115 (97.6%) in 2013, 189 754 (97.4%) in 2015, and 184 876 (94.9%) in 2018.^14^ Respondents aged 18-69 years were included in the present study as the 2004 survey only invited residents within this age range. The participants provided written informed consent.

### Data collection

Trained and qualified health professionals administered questionnaire based interviews from local health facilities (eg, hospitals, clinics, and centres for disease control and prevention), to collect data on personal, socioeconomic status, lifestyle behaviours, history of major chronic diseases, and prescribed drug use. In each survey round, blood pressure was measured in all respondents—three times successively with a one minute interval between measurements, using mercury sphygmomanometers in 2004 and electronic blood pressure monitors from 2007 (Omron, Dalian, China). The monitors were calibrated and tested according to standardprotocol. The average of the last two readings was used for analyses. Quality control was performed by national, provincial, and local designated staff according to a standard survey protocol. For most respondents, interviews, physical measurements, and biochemical sample collections were conducted at a community health centre in the sampled villages or residential areas. Those who did not participate in the survey on the due date received home visits; those who could not be reached after three invites were considered non-respondents.

### Outcome

Hypertension was defined as a systolic blood pressure of ≥140 mm Hg or diastolic blood pressure of ≥90 mm Hg or receiving drugs for hypertension. Awareness was calculated as the percentage of participants with hypertension who responded “Yes” to the question “Have you ever been told by a doctor or other healthcare professional that you had high blood pressure?” Treatment was calculated as the percentage of participants with hypertension who self-reported taking at least one prescribed antihypertensive drug for the management of hypertension. Control was calculated as the percentage of participants with hypertension who had a systolic blood pressure of <140 mm Hg and diastolic blood pressure of <90 mm Hg when measured in the survey. We also reported the proportion of adults with undiagnosed or untreated hypertension with a systolic blood pressure of ≥160 mm Hg or diastolic blood pressure of ≥100 mm Hg.

### Statistical analysis

The sample design in all analyses incorporated stratification, clustering, and sample weights that were computed as the product of original sampling weights, non-response weights, and post-stratification weights. To ensure overall prevalence estimates were comparable across the six rounds of survey, we standardised age specific results according to the 2010 China census population, to account for changes over time in age structure.^17^ For comparison, we also provided sample prevalence without standardisation. In addition, we estimated the absolute number of individuals with hypertension using post-stratification weights based on the United Nations population estimates of specific survey years,^18^ known as the Horvitz-Thompson estimator,^19^ to reflect the burden on the health system. We calculated prevalence, rates of awareness, treatment, and control, and mean blood pressure by sex, age, urban or rural residence, education, geographical region, body mass index, and central obesity. In the subgroups other than age, we calculated age adjusted rates by modelling design based multivariable logistic regression in *surveylogistic* procedure of SAS system with “lsmeans” statement (see supplement 2).^20^ We conducted preliminary analyses to assess whether trends in prevalence, awareness, treatment, and control rates by fitting linear, quadratic, cubic, and penalised cubic spline models. We further performed Welch’s *t* test for comparisons of annual absolute changes before and after 2010 to assess potential impacts on trends in prevalence of hypertension and management of national public health programmes related to hypertension prevention and control introduced around 2010. The annual absolute changes in prevalence were calculated as the absolute difference in prevalence between the start and end years divided by total number of years covered. See supplement 2 for further details of statistical analyses. We performed all analyses in SAS (version 9.4, SAS Institute, Cary, NC) and R (4.0.1).

### Patient and public involvement

This study used data from six rounds of the China Chronic Disease and Risk Factor Surveillance that were not specifically designed for the current study’s aim of evaluating the impact of the national public health programmes that were started in the late 2000s, by assessing the trend in hypertension prevalence and management. Therefore, we did not involve patients in setting the research question or in the design and implementation of the study.

## Results

The study included a total of 642 523 community dwelling adults aged 18-69 years (30 501 in 2004, 47 353 in 2007, 90 491 in 2010, 156 836 in 2013, 162 293 in 2015, and 155 049 in 2018; see supplementary fig 1). More women (53-58%) were recruited in each survey than men. Proportions of people older than 60 years, urban residents, and those with overweight, obesity, and central obesity gradually increased across the study period. The distributions for education and region were similar across all survey years (supplementary table 1).

### Hypertension prevalence

**Fig 1:**
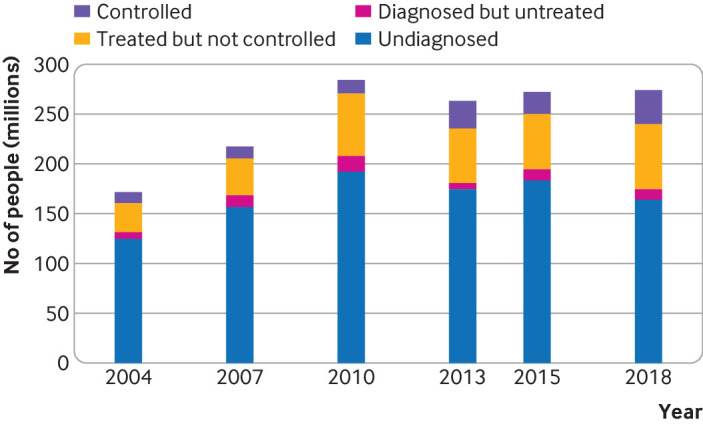
Trends in absolute burden of hypertension among adults aged 18-69 years in China, 2004-18. The absolute number was calculated based on United Nations population estimations for China in 2004, 2007, 2010, 2013, 2015, and 2018

The prevalence of hypertension in the study sample increased from 24.9% (95% confidence interval 24.4% to 25.3%) to 38.1% (37.8% to 38.3%) during 2004-18 (table 1). The standardised prevalence of hypertension increased from 20.8% (19.0% to 22.5%) in 2004 to 29.6% (27.8% to 31.3%) in 2010 (P<0.001), then decreased to 24.7% (23.2% to 26.1%) in 2018 (P<0.001 from 2010 to 2018) (table 1 and table 2). The absolute annual decline in prevalence of hypertension among women (−0.83 percentage points, 95% confidence interval −1.13 to −0.52) in 2010-18 was more than twice that of men (−0.40 percentage points, −0.73 to −0.07). The prevalence was higher in rural than urban areas in 2013 (P=0.04), although the differences were not statistically significant in other survey years. In China, 274 million (95% confidence interval 238 to 311 million) adults aged 18-69 years had hypertension in 2018 (fig 1), which was an increase from 172 million (135 to 208 million) in 2004 but a decline from the peak of 284 million (237 to 331 million) in 2010.

**Table 1:** Trends in crude and standardised rates for prevalence, awareness, treatment, and control of hypertension among men and women aged 18-69 years in China, by year of survey

	Survey year					
	2004	2007	2010	2013	2015	2018
**Crude rate (95% CI)**							
Overall:							
Hypertension	24.9 (24.4 to 25.3)	28.8 (28.4 to 29.3)	34.8 (34.5 to 35.2)	33.1 (32.8 to 33.3)	36.6 (36.4 to 36.8)	38.1 (37.8 to 38.3)	
Awareness	30.8 (29.8 to 31.9)	33.2 (32.4 to 34.0)	35.8 (35.2 to 36.3)	37.8 (37.4 to 38.2)	37.6 (37.2 to 38.0)	45.7 (45.3 to 46.1)	
Treatment	26.1 (25.1 to 27.1)	26.8 (26.1 to 27.6)	30.3 (29.8 to 30.8)	34.5 (34.1 to 34.9)	33.6 (33.2 to 33.9)	41.9 (41.5 to 42.3)	
Control	7.0 (6.4 to 7.6)	6.1 (5.7 to 6.5)	5.8 (5.5 to 6.1)	10.7 (10.4 to 11.0)	9.4 (9.2 to 9.7)	13.7 (13.4 to 14.0)	
Men:							
Hypertension	26.5 (25.7 to 27.2)	28.8 (28.2 to 29.4)	36.5 (36.0 to 37.0)	34.9 (34.5 to 35.2)	38.8 (38.5 to 39.2)	40.9 (40.5 to 41.3)	
Awareness	25.0 (23.6 to 26.5)	30.0 (28.8 to 31.1)	32.4 (31.7 to 33.2)	34.6 (34.0 to 35.2)	33.8 (33.3 to 34.4)	41.8 (41.2 to 42.4)	
Treatment	20.1 (18.7 to 21.4)	23.3 (22.2 to 24.3)	26.1 (25.4 to 26.8)	30.8 (30.2 to 31.4)	29.5 (28.9 to 30.0)	37.6 (37.1 to 38.2)	
Control	5.5 (4.7 to 6.2)	5.3 (4.8 to 5.9)	5.2 (4.8 to 5.5)	9.7 (9.3 to 10.1)	8.6 (8.2 to 8.9)	12.3 (11.9 to 12.7)	
Women:							
Hypertension	23.6 (23.0 to 24.2)	28.9 (28.3 to 29.5)	33.5 (33.0 to 33.9)	31.8 (31.4 to 32.1)	34.7 (34.4 to 35.0)	35.9 (35.6 to 36.2)	
Awareness	35.9 (34.4 to 37.4)	36.1 (35.0 to 37.2)	38.8 (38.1 to 39.6)	40.4 (39.9 to 41.0)	41.1 (40.6 to 41.7)	49.1 (48.5 to 49.6)	
Treatment	31.5 (30.0 to 32.9)	30.0 (28.9 to 31.0)	34.2 (33.4 to 34.9)	37.5 (36.9 to 38.0)	37.5 (36.9 to 38.0)	45.7 (45.2 to 46.3)	
Control	8.4 (7.5 to 9.2)	6.8 (6.2 to 7.4)	6.4 (6.0 to 6.8)	11.5 (11.1 to 11.9)	10.3 (9.9 to 10.6)	15.0 (14.6 to 15.4)	
Urban:							
Hypertension	26.5 (25.7 to 27.3)	29.2 (28.6 to 29.9)	35.0 (34.5 to 35.4)	33.2 (32.9 to 33.6)	36.1 (35.8 to 36.5)	38.4 (38.0 to 38.7)	
Awareness	39.7 (37.9 to 41.4)	41.4 (40.0 to 42.7)	39.5 (38.7 to 40.4)	44.1 (43.5 to 44.8)	42.5 (41.9 to 43.1)	49.0 (48.4 to 49.6)	
Treatment	33.1 (31.4 to 34.7)	33.8 (32.5 to 35.0)	33.9 (33.1 to 34.7)	40.9 (40.2 to 41.5)	38.8 (38.2 to 39.4)	45.7 (45.1 to 46.3)	
Control	9.3 (8.3 to 10.3)	8.9 (8.1 to 9.6)	8.2 (7.7 to 8.7)	14.7 (14.3 to 15.2)	12.7 (12.3 to 13.1)	17.4 (17.0 to 17.9)	
Rural:							
Hypertension	23.8 (23.2 to 24.4)	28.6 (28.1 to 29.1)	34.8 (34.4 to 35.2)	32.9 (32.6 to 33.2)	37.0 (36.7 to 37.3)	37.8 (37.5 to 38.1)	
Awareness	24.7 (23.5 to 26.0)	27.9 (27.0 to 28.9)	33.3 (32.6 to 34.0)	33.3 (32.8 to 33.8)	34.0 (33.5 to 34.5)	42.9 (42.4 to 43.4)	
Treatment	21.3 (20.1 to 22.5)	22.3 (21.4 to 23.2)	27.9 (27.3 to 28.6)	30.0 (29.4 to 30.5)	29.8 (29.3 to 30.2)	38.8 (38.2 to 39.3)	
Control	5.5 (4.8 to 6.1)	4.3 (3.9 to 4.8)	4.2 (3.9 to 4.5)	7.8 (7.5 to 8.1)	7.1 (6.8 to 7.3)	10.6 (10.2 to 10.9)	
Standardised rate (95% CI)*							
Overall:							
Hypertension	20.8 (19.0 to 22.5)	24.1 (22.2 to 26.0)	29.6 (27.8 to 31.3)	25.1 (24.1 to 26.1)	25.2 (23.6 to 26.7)	24.7 (23.2 to 26.1)	
Awareness	30.8 (28.0 to 33.6)	31.6 (29.0 to 34.2)	33.7 (31.8 to 35.6)	31.7 (30.0 to 33.3)	32.6 (31.0 to 34.2)	38.3 (36.3 to 40.4)
Treatment	25.9 (23.5 to 28.4)	25.5 (23.1 to 27.9)	27.9 (26.2 to 29.6)	28.7 (27.1 to 30.3)	28.5 (27.0 to 30.1)	34.6 (32.6 to 36.7)
Control	7.1 (6.0 to 8.2)	6.7 (5.4 to 7.9)	5.5 (4.8 to 6.2)	8.9 (7.9 to 9.8)	8.8 (7.9 to 9.7)	12.0 (10.6 to 13.4)
Men:						
Hypertension	22.4 (20.3 to 24.4)	24.2 (22.3 to 26.2)	32.2 (30.3 to 34.0)	27.4 (26.3 to 28.5)	28.4 (26.6 to 30.2)	29.0 (27.1 to 30.9)
Awareness	25.7 (23.0 to 28.4)	29.2 (26.4 to 31.9)	30.9 (29.0 to 32.7)	29.0 (27.3 to 30.8)	29.2 (27.5 to 30.9)	34.3 (31.9 to 36.8)
Treatment	20.5 (18.2 to 22.8)	22.4 (19.8 to 25.0)	24.2 (22.5 to 25.8)	25.7 (24.0 to 27.3)	24.7 (23.2 to 26.3)	30.3 (28.0 to 32.6)
Control	5.6 (4.5 to 6.8)	6.0 (4.7 to 7.4)	4.9 (4.2 to 5.5)	8.3 (7.3 to 9.2)	7.5 (6.6 to 8.4)	10.3 (9.0 to 11.6)
Women:						
Hypertension	19.2 (17.6 to 20.8)	24.0 (21.9 to 26.1)	26.8 (25.0 to 28.6)	22.6 (21.6 to 23.7)	21.8 (20.4 to 23.3)	20.2 (18.6 to 21.8)
Awareness	37.0 (33.6 to 40.3)	34.2 (31.2 to 37.2)	37.2 (35.0 to 39.5)	35.0 (33.1 to 36.8)	37.2 (35.3 to 39.0)	44.2 (42.1 to 46.3)
Treatment	32.5 (29.6 to 35.4)	28.8 (26.3 to 31.3)	32.5 (30.5 to 34.6)	32.4 (30.6 to 34.2)	33.7 (31.8 to 35.5)	41.1 (39.0 to 43.3)
Control	8.8 (7.3 to 10.2)	7.3 (5.9 to 8.8)	6.3 (5.4 to 7.2)	9.6 (8.5 to 10.6)	10.6 (9.4 to 11.7)	14.5 (12.3 to 16.7)
Urban:						
Hypertension	20.5 (17.9 to 23.0)	23.0 (20.7 to 25.4)	28.9 (26.4 to 31.3)	23.7 (22.2 to 25.2)	23.9 (21.4 to 26.3)	24.9 (22.8 to 27.1)
Awareness	36.3 (31.9 to 40.7)	37.0 (33.0 to 41.1)	35.6 (32.5 to 38.7)	37.5 (34.6 to 40.5)	36.3 (33.9 to 38.7)	38.9 (35.6 to 42.2)
Treatment	29.9 (26.0 to 33.8)	30.1 (26.3 to 34.0)	29.6 (26.7 to 32.4)	34.4 (31.5 to 37.2)	32.3 (29.9 to 34.6)	35.6 (32.4 to 38.8)
Control	8.4 (6.7 to 10.2)	8.7 (6.5 to 10.8)	7.1 (5.9 to 8.3)	12.9 (11.0 to 14.8)	11.3 (9.8 to 12.9)	14.0 (11.6 to 16.4)
Rural:						
Hypertension	21.2 (18.9 to 23.4)	25.4 (22.5 to 28.3)	30.4 (27.9 to 32.9)	25.9 (24.6 to 27.2)	26.7 (25.3 to 28.2)	24.4 (22.4 to 26.3)
Awareness	24.5 (22.5 to 26.6)	25.8 (23.7 to 27.9)	31.5 (29.4 to 33.6)	28.4 (26.6 to 30.2)	28.7 (26.9 to 30.4)	37.6 (35.4 to 39.8)
Treatment	21.4 (19.6 to 23.2)	20.6 (18.7 to 22.5)	26.1 (24.2 to 27.9)	25.5 (23.8 to 27.2)	24.6 (22.9 to 26.3)	33.5 (31.2 to 35.7)
Control	5.5 (4.6 to 6.4)	4.5 (3.6 to 5.3)	3.8 (3.2 to 4.4)	6.6 (5.8 to 7.4)	6.1 (5.3 to 7.0)	9.5 (8.1 to 11.0)

CI=confidence interval.

* Rates weighted to 2010 census population in China.

**Table 2:** Absolute annual change (percentage points) in standardised rates for prevalence, awareness, treatment, and control of hypertension in China during 2004-10, 2010-18, and 2004-18

	2004-10			2010-18			2004-18		P value*
	Annual change (95% CI)	P value		Annual change (95% CI)	P value		Annual change (95% CI)	P value	
**Overall**									
Hypertension	1.47 (1.05 to 1.88)	<0.001		−0.61 (−0.90 to −0.33)	<0.001		0.28 (0.12 to 0.44)	<0.001	<0.001
Awareness	0.48 (−0.08 to 1.05)	0.09		0.58 (0.23 to 0.92)	0.001		0.54 (0.29 to 0.78)	<0.001	0.79
Treatment	0.33 (−0.16 to 0.83)	0.19		0.84 (0.50 to 1.17)	<0.001		0.62 (0.39 to 0.85)	<0.001	0.10
Control	−0.27 (−0.48 to −0.05)	0.02		0.81 (0.62 to 1.01)	<0.001		0.35 (0.22 to 0.48)	<0.001	<0.001
**Men**									
Hypertension	1.63 (1.17 to 2.09)	<0.001		−0.40 (−0.73 to −0.07)	0.02		0.47 (0.27 to 0.67)	<0.001	<0.001
Awareness	0.87 (0.32 to 1.41)	0.002		0.43 (0.04 to 0.81)	0.03		0.61 (0.35 to 0.87)	<0.001	0.19
Treatment	0.62 (0.14 to 1.09)	0.01		0.76 (0.41 to 1.12)	<0.001		0.70 (0.47 to 0.93)	<0.001	0.63
Control	−0.12 (−0.34 to 0.10)	0.30		0.68 (0.49 to 0.86)	<0.001		0.34 (0.21 to 0.46)	<0.001	<0.001
**Women**									
Hypertension	1.27 (0.87 to 1.67)	<0.001		−0.83 (−1.13 to −0.52)	<0.001		0.07 (−0.09 to 0.23)	0.39	<0.001
Awareness	0.03 (−0.64 to 0.71)	0.92		0.88 (0.49 to 1.26)	<0.001		0.51 (0.23 to 0.80)	<0.001	0.03
Treatment	0.00 (−0.59 to 0.59)	>0.99		1.08 (0.70 to 1.45)	<0.001		0.61 (0.36 to 0.87)	<0.001	0.003
Control	−0.42 (−0.70 to −0.13)	0.004		1.03 (0.73 to 1.32)	<0.001		0.41 (0.22 to 0.60)	<0.001	<0.001
**Urban**									
Hypertension	1.40 (0.81 to 1.99)	<0.001		−0.50 (−0.91 to −0.09)	0.02		0.31 (0.08 to 0.55)	0.01	<0.001
Awareness	−0.12 (−1.01 to 0.78)	0.80		0.41 (−0.15 to 0.98)	0.15		0.19 (−0.21 to 0.58)	0.35	0.33
Treatment	−0.05 (−0.86 to 0.76)	0.90		0.75 (0.21 to 1.29)	0.006		0.41 (0.05 to 0.77)	0.03	0.11
Control	−0.22 (−0.57 to 0.14)	0.23		0.86 (0.53 to 1.20)	<0.001		0.40 (0.19 to 0.61)	<0.001	<0.001
**Rural**									
Hypertension	1.53 (0.97 to 2.09)	<0.001		−0.75 (−1.15 to −0.35)	<0.001		0.23 (0.02 to 0.44)	0.04	<0.001
Awareness	1.17 (0.68 to 1.66)	<0.001		0.76 (0.38 to 1.14)	<0.001		0.94 (0.72 to 1.15)	<0.001	0.20
Treatment	0.78 (0.35 to 1.21)	<0.001		0.93 (0.56 to 1.29)	<0.001		0.86 (0.66 to 1.07)	<0.001	0.62
Control	−0.28 (−0.46 to −0.10)	0.002		0.71 (0.52 to 0.91)	<0.001		0.29 (0.16 to 0.41)	<0.001	<0.001

CI=confidence interval.

^*^ P value for difference in annual changes for 2004-10 *v* 2010-18.

**Fig 2:**
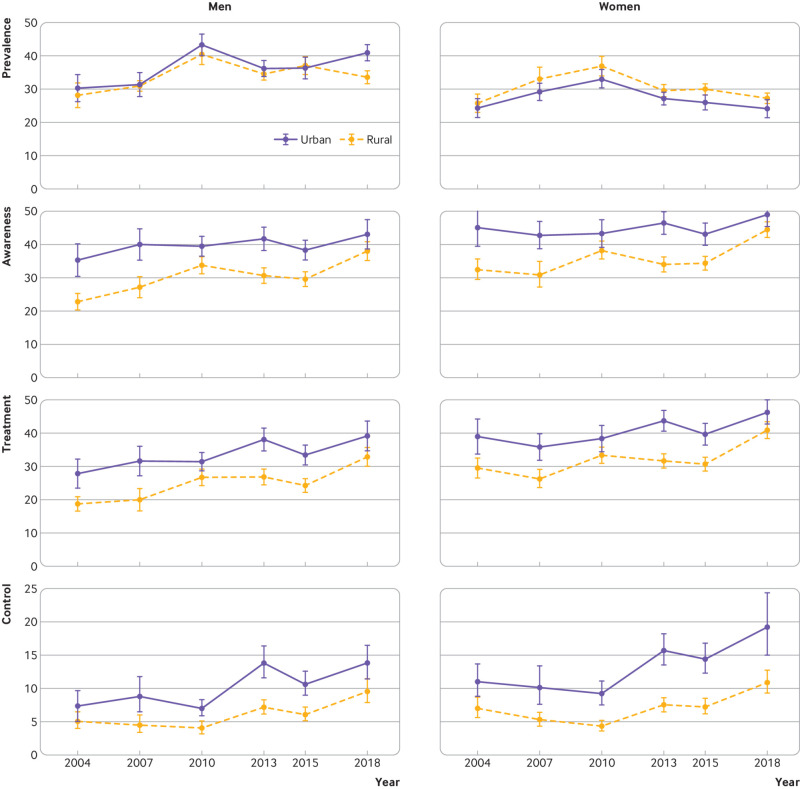
Trends in hypertension prevalence, awareness, treatment, and control rates in urban and rural areas among men and women aged 18-69 years in China, 2004-18. All rates were estimated using multivariable logistic regression containing age, survey year, sex, residence, and two way and three way interaction terms of survey year, sex, and residence. Error bars indicate 95% confidence intervals with consideration of complex sample design

**Fig 3:**
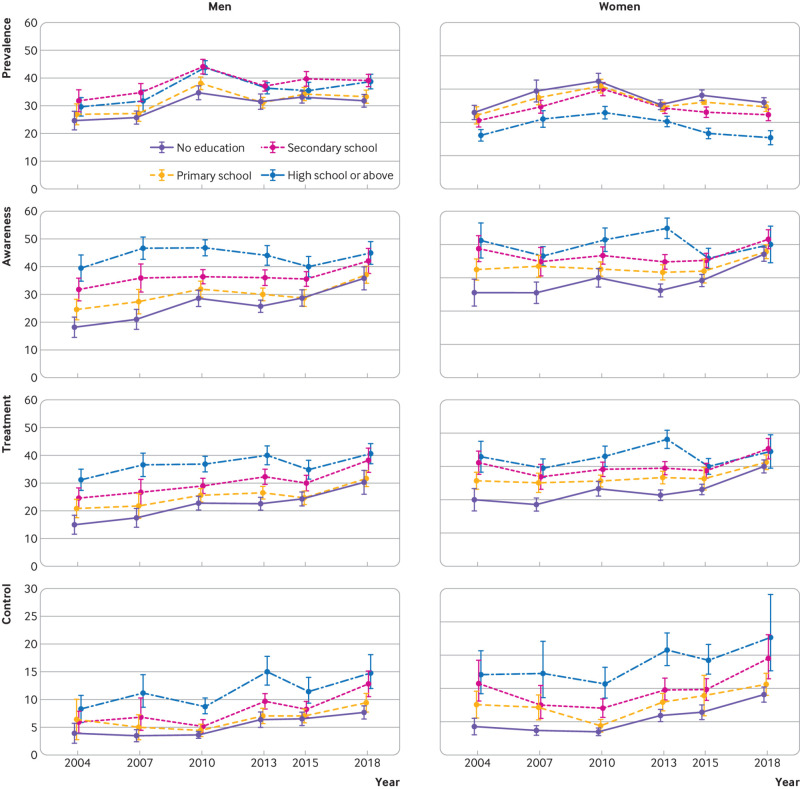
Trends in hypertension prevalence, awareness, treatment, and control rates by education group among men and women aged 18-69 years in China, 2004-18. All rates were estimated using multivariable logistic regression containing age, survey year, sex, education, and two way and three way interaction terms of survey year, sex, and education. Error bars indicate 95% confidence intervals with consideration of complex sample design

**Fig 4:**
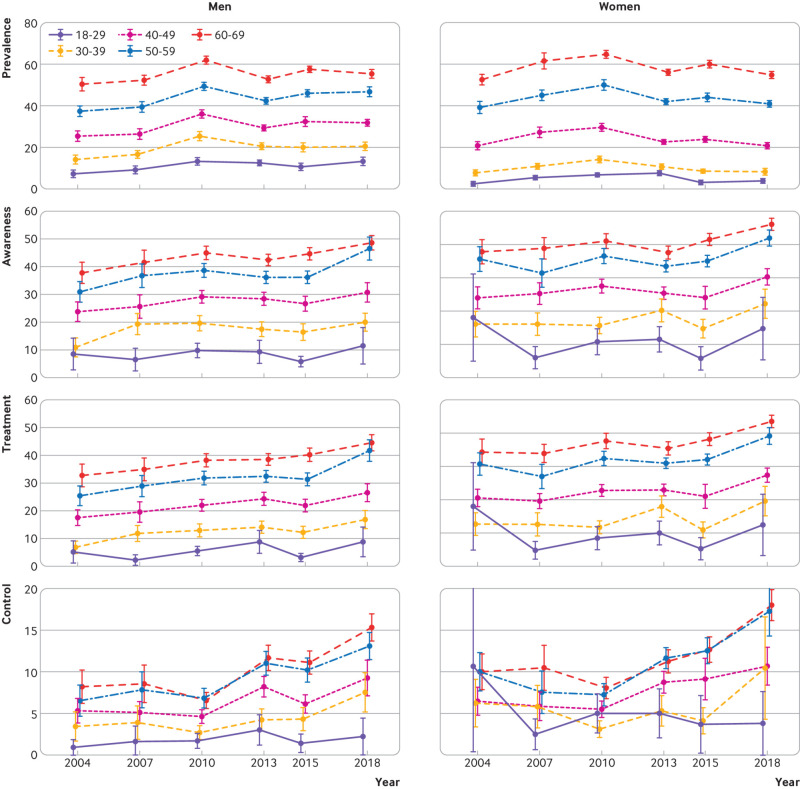
Trends in standardised hypertension prevalence, awareness, treatment, and control rates by age groups among men and women in China, 2004-18. All rates were weighted to the 2010 China population. Error bars indicate 95% confidence intervals with consideration of complex sample design

The trends in hypertension prevalence were largely similar in urban and rural areas for both sexes (fig 2 and supplementary table 2). Women in rural areas had a somewhat higher prevalence of hypertension than their urban counterparts across all survey years, whereas prevalence was similar in men from urban and rural areas, except in 2018 (P>0.05 during 2004-15, P=0.02 in 2018). Less educated women had a higher prevalence of hypertension than women with better education attainment in all survey years, but the education gradient was mixed among men (fig 3 and supplementary table 3). For both sexes there were similar trends in hypertension prevalence across age groups (fig 4). The prevalence in women was lower than in men among adults younger than 50 years in all survey years, but higher than in men older than 60 years, except in 2018 (supplementary table 4). Men and women in the north region generally had the highest prevalence and those in the south region had the lowest (supplementary fig 2 and supplementary table 5). Across regions, about a twofold difference in hypertension prevalence was observed in all survey years.

In each survey year for both sexes, the prevalence of hypertension approximately doubled among those with normal body mass index, tripled in overweight adults, and quadrupled or quintupled in obese adults compared with underweight adults (supplementary fig 2 and supplementary table 6). The prevalence for both men and women with central obesity was approximately twice as high as their counterpart without central obesity in each survey year (supplementary fig 2 and supplementary table 7).

### Awareness, treatment, and control of hypertension

Awareness, treatment, and control of hypertension were low throughout the study period. Modest improvements have occurred since 2004, with standardised rates of awareness increasing from 30.8% (28.0% to 33.6%) to 38.3% (36.3% to 40.4%), treatment from 25.9% (23.5% to 28.4%) to 34.6% (32.6% to 36.7%), and control from 7.1% (6.0% to 8.2%) to 12.0% (10.6% to 13.4%) (all P<0.001) (table 1 and table 2). Among men, the rates of awareness, treatment, and control increased annually by 0.61 (0.35 to 0.87), 0.70 (0.47 to 0.93), and 0.34 (0.21 to 0.46) percentage points from 2004 to 2018 (all P<0.001) (table 2). Among women, the annual absolute change in rates of awareness, treatment, and control during 2010-18 were statistically higher than during 2004-10 (P=0.03, P=0.003, P<0.001, respectively). The annual changes in awareness and treatment in rural areas was greater than those in urban areas (awareness: 0.94 (0.72 to 1.15) *v* 0.19 (−0.21 to 0.58) percentage points per year, P=0.001; treatment: 0.86 (0.66 to 1.07) *v* 0.41 (0.05 to 0.77) percentage points per year, P=0.03) (table 2). Yet control in urban areas annually increased at a higher pace than in rural areas, without statistical significance (0.40 (0.19 to 0.61) *v* 0.29 (0.16 to 0.41) percentage points per year, P=0.36).

In all survey years studied, both men and women in urban areas had better awareness, treatment, and control of hypertension than their rural counterparts (fig 2 and supplementary table 2). The urban-rural gaps in awareness and treatment narrowed between 2004 and 2018, but the gaps for control remained unchanged owing to a widening urban-rural gap in the control rate among treated individuals (see supplementary table 8). Rates improved for all education subgroups from 2004 to 2018, but higher education groups showed smaller progress (fig 3 and supplementary table 3). Similar trends were seen across age groups (fig 4 and supplementary table 4).

Awareness, treatment, and control of hypertension improved between 2004 and 2018 across all regions of China except the south region, but regional and provincial variation persisted (supplementary table 5 and supplementary fig 3). The provincial difference in awareness, treatment, and control is partly correlated with per capita GDP (supplementary fig 4). Better awareness and treatment were consistently found in men and women with obesity or central obesity than their counterparts without obesity in all survey years (supplementary tables 6 and 7), but the same advantage was not seen for control. When we used an alternative definition of hypertension (130/80 mm Hg), results for hypertension prevalence were similar to the main analysis (supplementary table 9).

In 2018, an estimated 240 million (95% confidence interval 215 to 264 million) adults aged 18-69 years with hypertension in China had inadequately controlled blood pressure; among them, 164 million (141 to 186 million) were not aware of their condition and a further 10 million (9 to 12 million) were not receiving appropriate treatment. Despite a slight decrease in blood pressure, hypertension >160/100 mm Hg was not diagnosed or treated in more than 10% of men and women in 2018 (supplementary table 10).

## Discussion

Using information from six rounds of a large national survey during 2004-18, our study found that although the prevalence of hypertension might have decreased moderately in China since 2010, awareness, treatment, and control remained low throughout the period. Although under-detection of hypertension was universally observed across all subgroups, large inequalities persisted in hypertension prevalence, awareness, treatment, and control between urban and rural areas, men and women, geographical regions, and socioeconomic groups.

### Comparison with previous studies

A few studies have reported the national prevalence of hypertension at a single time point in China; the prevalence estimated in our study was generally consistent with the findings of those studies for similar time points.^11^ ^12^ ^13^ Specifically, the 2007-08 China National Diabetes and Metabolic Disorders Study reported that 27% of Chinese adults aged 20 years or older from 14 provinces had hypertension, compared with 24% for 18-69 years old in 2007 in our study.^11^ In the China National Survey of Chronic Kidney Disease, the adjusted prevalence of hypertension was reported as 30% for adults aged 18 years or older in 2009-10, which was the same as our findings for 2010 (30% for those aged ≥20 years).^12^ In a more recent nationwide study involving 451 755 residents from 31 provinces in mainland China, the reported prevalence of hypertension (23%) in 2012-15 was slightly lower than our findings (25% in 2013, 25% in 2015) possibly because the study used a different definition for hypertension to ours (ie, systolic blood pressure ≥140 mm Hg, diastolic blood pressure ≥90 mm Hg, or use of antihypertensive drugs within two weeks).^13^ Several subnational studies reported trends in hypertension prevalence and management in parts of China,^21^ ^22^ ^23^ but few had assessed trends for the whole of China. A nationwide study used only post-2010 data on adults aged 45 years or older and reported that the weighted prevalence of hypertension increased from 41% in 2011 to 43% in 2013 and then declined to 42% in 2015, with improvements in diagnosis, treatment, and control of hypertension.^24^ The only study that reported nationwide trends in hypertension prevalence involving pre-2010 and post-2010 time points in China was a longitudinal cohort in the China Health and Nutrition Study, which used samples from 15 provinces that are not nationally representative and had a total sample size of 72 452 over nine rounds of surveys, much smaller than ours.^25^ It reported that the age standardised prevalence and its awareness, treatment, and control rates among adults aged 20-79 years increased with fluctuations from 1991 to 2015 (from 15% in 1991 to 26% in 2015). A recent meta-analysis pooled 18 national and non-national studies that reported hypertension prevalence in China from 1959 to 2018, including three rounds of the China Chronic Disease and Risk Factor Surveillance survey.^26^ It found a high heterogeneity (I^2^=99.9%) in the included studies, partly because these studies differed in age ranges and methods for age standardisation, and it reported that hypertension prevalence increased between 2004 and 2010 and declined in 2011 before continuing to increase through 2018.

### Strengths and limitations of this study

Using consecutive, large scale, nationally representative surveys that adopted broadly consistent protocols, we provide information on the long term and recent trends in hypertension prevalence, awareness, treatment, and control in China in the past 15 years. The large sample sizes and regional representativeness also allowed reliable subgroup analyses of trends and variations. As with most long running national health surveys, our study also has limitations. Mercury sphygmomanometers were used in the 2004 survey, whereas electronic monitors were used in all other years; nonetheless, all electronic devices (Omron HEM-770A in 2007, HEM-7071 in 2010, and HBP-1300 in 2013, 2015, and 2018) had passed the accuracy standards and inspection of both the Association for the Advancement of Medical Instruments and the British Hypertension Society. In addition, we used self-reported status of antihypertensive drug use and history of hypertension diagnosis, which may be subject to recall bias. Previous studies showed that such self-reported drug and diagnosis history are in good agreement with medical records.^27^ ^28^ We did not collect information on whether participants had been screened for hypertension or had sought care for hypertension. As with all population health surveillance efforts, our results might be affected by non-responses of survey participants and how non-responses varied across different surveys. Nonetheless, the response rate was high in all survey rounds, ranging from 90.5% in 2010 to 99.1% in 2007. In the 2010 survey, non-response rate showed no statistically significant association between hypertension prevalence and population characteristics, including age, marital status, education, and self-reported diabetes at survey county or district level. Biased estimation, however, might be introduced by non-response if differences existed in the characteristics that were associated with hypertension status between participants and those who refused to respond. Even after the adjustment in the weighting process for household and individual non-response, over-estimation or under-estimation of prevalence might persist and account for some of the observed patterns or trends. Despite independent sampling in each round of the China Chronic Disease and Risk Factor Surveillance survey, a small proportion of individuals were repeatedly included during 2004-18, which might contribute to a less precise estimation of hypertension prevalence and management. Owing to the limitation of having only six time points of surveys, we were unable to estimate the linear and non-linear trends robustly.

### Interpretations and implications

Despite aging of the population, we found decreases in the number of individuals aged between 18 and 69 years with hypertension since 2010, mainly because of the decrease in prevalence of hypertension in the same period in most ages (fig 4). The decline in prevalence coincided with the introduction of several national health promotion programmes,^29^ which aimed to promote healthier lifestyles and risk reduction and might have played a role in the observed decline. Some improvement in diet was observed from 2002 and 2012, including an increase in fruit consumption and decrease in intake of refined carbohydrates and total energy.^30^ Additionally, salt intake has decreased since the 2000s,^31^ although levels of sodium intake in China still exceed recommended limits and remain among the highest in the world.^32^ The mild decrease in salt intake also failed to explain the increase in hypertension prevalence we observed from 2004 to 2010. As one of the major risk factors for hypertension,^33^ the increase in obesity prevalence slowed down similarly from 2010.^16^

Despite modest improvements in the management of hypertension in China, control was unsatisfactory. The control rate we found in China in 2018 (10-15%) was much lower than in high income countries in North America and Europe, as well as in South Korea and Japan (30-60%).^5^ Some countries with similar per capita national income as China’s, such as Costa Rica and Mexico, achieved hypertension control twice that of what we found in China, showing the feasibility of achieving better control in resource limited health systems.^5^

Nearly 60% of people with hypertension in China never received a diagnosis (supplementary fig 5). Therefore, better detection of people with hypertension is an important task. Experience from countries that have substantially improved the detection of hypertension typically involved improving the coverage of health insurance and regular blood pressure checks in health facilities according to guidelines.^34^ ^35^ ^36^ In China, the National Health and Family Planning Commission published guidance in 2009 that included mandatory blood pressure measurement for people older than 35 years when visiting hospital for the first time.^37^ Our findings, however, suggest poor implementation of guidance in practice.

Less than one third of people treated for hypertension in China achieved control (supplementary fig 5), therefore improving detection alone is unlikely to lead to sufficient improvements in hypertension control. At least four additional measures are needed. Firstly, the availability and affordability of multiple antihypertensive drug classes are essential to improve hypertension control.^38^ Poor access to multiple drugs and consequent underuse might have been an important factor behind the poor control we observed.^39^ ^40^ Secondly, increasing the frequency of clinical visits could improve both adherence to antihypertensive treatment and hypertension control.^41^ Current guidelines in China recommend that patients with hypertension visit their general practitioners at least once every 1-3 months,^42^ but several subnational surveys in China found that only 60% of patients with hypertension visited their doctors frequently enough.^41^ ^43^ Thirdly, besides healthcare facilities, communities and workplaces have also been important settings for implementing broad management programmes for hypertension prevention and management.^44^ ^45^ Fourthly, improving the use of home blood pressure monitoring could help improve patient adherence to treatment and thereby reduce high blood pressure.^46^ ^47^ With emerging technology and availability of smart phones, mHealth tools and devices such as wearable blood pressure monitors might help better self-management of hypertension, improve the reach of healthcare services, and promote healthy behaviour in users.^48^ ^49^

Involvement of multidisciplinary staff in primary care is needed to enhance the synergy of multiple interventions that are being implemented. In the 1980s, the Capital Steel and Iron Company cardiovascular intervention programme, embedded in the local primary care system, achieved treatment and control rates as high as 98% and 72%.^50^ With rapid urbanisation and the rise and expansion of large state owned hospitals in cities, however, the role of primary care in hypertension management across China has diminished since the 1980s. There is a critical shortage of qualified general practitioners in China, particularly in rural areas,^51^ ^52^ and knowledge of hypertension management among existing doctors is unsatisfactory,^53^ leading to poor implementation of hypertension guidelines. Despite the huge investment since the launch of National Basic Public Health Servicein 2009, financial and non-financial incentive mechanisms to improve quality of care delivered by general practitioners are still lacking.^54^ Capacity building and system strengthening in primary care are necessary throughout China for the large scale implementation of proven actions for improving hypertension control, such as opportunistic screening, providing public awareness programmes, and training doctors to reduce clinical inertia.

We also found that the urban-rural gap in hypertension control persisted despite a narrowing gap in awareness and treatment. The unified medical insurance scheme for urban and rural residents, rolled out in 2014 to replace the previously fragmented schemes, gradually reduced the gaps in healthcare utilisation and subsequently hypertension detection and treatment between rural and urban residents.^55^ A study also reported more progress in the proportion of antihypertensive drug use in rural areas than in urban areas from 2000 to 2011.^56^ Compared with people in urban areas, however, people in rural areas still have poorer adherence to treatment^57^ and access to healthcare,^58^ and primary care doctors have poorer adherence to guidelines,^59^ leading to the persisting gap in control between rural and urban areas. Therefore, it is important to build on the healthcare reform in rural areas to provide health education programmes for patients and doctors to encourage adherence to treatment.

We found that the prevalence of hypertension was inversely associated with education level among women, but the association was more mixed among men, consistent with studies from other countries.^60^ ^61^ ^62^ The sex specific education gradient in hypertension was similar to that observed for body mass index in China.^16^ The difference in sex specific education gradient in hypertension might be attributed to women with higher education being both better informed of health information and more apt to modify their lifestyles than men with higher education, compared with their counterparts with lower education.^63^

### Conclusions

Despite moderate improvements in the prevalence and management of hypertension among adults in China, coverage of hypertension treatment and control remains low, highlighting the need for primary care to become reinvolved in the management of hypertension. Efforts should aim to both scale-up the detection of hypertension and enhance control among patients receiving treatment. Interventions are also needed to reduce the burden of hypertension in rural areas.

**What is already known on this topic**

Hypertension is a major cause of mortality and morbidity in China
Since the late 2000s, China has started multiple nationwide programmes for the prevention and control of hypertension
Current estimates of contemporary long term trends in hypertension prevalence, awareness, treatment, and control in China were based on data without national representativeness before 2015

**What this study adds**

Based on six rounds of a nationally representative survey during 2004-18 in China, this study showed that after an initial increase the standardised prevalence of hypertension declined from 2010
The awareness, treatment, and control of hypertension remained low throughout the study period
In 2018, an estimated 274 million people aged 18-69 years had hypertension in China and in most (240 million) control was inadequate

## Data Availability

Individual participant data will not be made available publicly. For further detailed data access policy and procedure, contact jianceshi@ncncd.chinacdc.cn.

## Web extra

Extra material supplied by authors

Supplementary information: Supplement 1-4, supplementary figures 1-7, and supplementary tables 1-12

## Notes

Contributors: MZ, YS, and BZ contributed equally to the paper as joint first authors. LW (wanglimin@ncncd.chinacdc.cn) and YL (yichongli.cvd@139.com) are joint corresponding authors. YW and ME are joint senior authors. MZ, YS, BZ, ME, LW, and YL developed the study concept and drafted analyses plan. MZ, ZH, ZZ, CL, XZ, LW, and YL collected data. MZ, YS, and BZ conducted analysis and prepared results. MZ, YS, BZ, ME, LW, and YL wrote the first draft of the paper. YW and ME provided critical review. All authors provided input into interpretation of results and contents of the paper. MZ and LW had full access to all of the data in the study and verified the data. MZ, LW, and YL are responsible for the integrity of the data, accuracy of the data analysis, and decision to submit the manuscript, and act as guarantors. The corresponding author attests that all listed authors meet authorship criteria and that no others meeting the criteria have been omitted.

The lead authors (LW and YL) affirm that the manuscript is an honest, accurate, and transparent account of the study being reported; that no important aspects of the study have been omitted; and that any discrepancies from the study as planned (and, if relevant, registered) have been explained.

Dissemination to participants and related patient and public communities: We plan to disseminate these findings to participants and the general public through online news websites, press conferences, and printed survey reports.

Provenance and peer review: Not commissioned; externally peer reviewed.
