# Supplementary material for: Prevalence, awareness, treatment, and control of hypertension in China, 2004-18: findings from six rounds of a national survey
Source: BMJ. 2023 Jan 11;380:e071952. doi: 10.1136/bmj-2022-071952 (PMC10498511; doi:10.1136/bmj-2022-071952)
Supplement: Supplementary file 1 — Supplementary information: Supplement 1-4, supplementary figures 1-7, and supplementary tables 1-12 [file zham071952.ww1.pdf]

Supplementary Materials to

Hypertension prevalence, awareness, treatment, and control in China, 2004-2018: findings from six rounds of a nationally representative surveys involving 642,523 participants

Authors:

Mei Zhang,[1] Yu Shi,[2] Bin Zhou,[3,4,5] Zhengjing Huang,[1] Zhenping Zhao,[1] Chun Li,[1] Xiao Zhang,[1] Guiyuan Han,[2] Ke Peng,[2] Xinhua Li,[1,6] Youfa Wang,[7] Majid Ezzati,[3,4,5] Limin Wang,[1] Yichong Li[2]

Affiliations:

[1]National Center for Chronic and Noncommunicable Disease Control and Prevention, Chinese Center for Disease Control and Prevention, Beijing 100050, China

[2]National Clinical Research Center for Cardiovascular Diseases, Heart Failure Ward, Fuwai Hospital Chinese Academy of Medical Sciences, Shenzhen, Guangdong Province, China

[3]Department of Epidemiology and Biostatistics, School of Public Health, Imperial College London, London, UK

[4]MRC Centre for Environment and Health, School of Public Health, Imperial College London, London, UK

[5]The Abdul Latif Jameel Institute for Disease and Emergency Analytics, School of Public Health, Imperial College London, London, UK

[6]People's Medical Publishing House, Beijing, China

[7]Global Health Institute, School of Public Health, Xi'an Jiaotong University, Xi'an, China

Correspondence: Limin Wang, MPH, National Center for Chronic and Noncommunicable Disease Control and Prevention, Chinese Center for Disease Control and Prevention, 27 Nanwei Road, Xicheng District, Beijing 100050, China; email: wanglimin@ncncd.chinacdc.cn.

Yichong Li, PhD, National Clinical Research Center for Cardiovascular Diseases, Fuwai Hospital Chinese Academy of Medical Sciences, 12 Langshan Road, Nanshan District, Shenzhen 518057, Guangdong Province, China; email: yichongli.cvd@139.com.

Table of contents

Supplement 1. Details of sampling scheme 4

Supplement 2. Further details on statistical analyses 7

Supplement 3. Further details on blood pressure monitors 9

Supplement 4. Mean BP and raised BP prevalence 10

Supplementary Fig 1. Sampling scheme of CCDRFS surveys 11

Supplementary Fig 2. Trends in adjusted hypertension prevalence by region, BMI and central obesity status among men and women from 2004 to 2018. 12

Supplementary Fig 3. The geographic variation in hypertension (A) prevalence, (B) awareness, (C) treatment and (D) control in China in 2018. 13

Supplementary Fig 4. Hypertension care cascade indicators by GDP per capita in different provinces of China in 2018. 14

Supplementary Fig 5. The hypertension care cascade in China in 2018. 15

Supplementary Fig 6. Proportion of participants with hypertension reaching each cascade step, stratified by sex, age group, and education in 2018. 16

Supplementary Fig 7. Linear and nonlinear trends of hypertension prevalence, awareness, treatment, and control with survey year. 17

Supplementary Table 1. Demographics characteristic of CCDRFS surveys 18

Supplementary Table 2. Trends in hypertension prevalence, awareness, treatment, and control rates in urban and rural areas 20

Supplementary Table 3. Trends in hypertension prevalence, awareness, treatment, and control rates by education 21

Supplementary Table 4. Trends in standardised hypertension prevalence, awareness, treatment, and control rates by age group 23

Supplementary Table 5. Trends in hypertension prevalence, awareness, treatment, and control rates by region 25

Supplementary Table 6. Trends in hypertension prevalence, awareness, treatment, and control rates by BMI groups 27

Supplementary Table 7. Trends in hypertension prevalence, awareness, treatment, and control rates by central obesity status 29

Supplementary Table 8. Trends in control rates among people under treatment for hypertension 30

Supplementary Table 9. Trends in standardised hypertension prevalence (130/80 mmHg or diagnosed) in overall and by subgroups 33

Supplementary Table 10. Proportion of adults with hypertension who had SBP ≥160 mmHg or DBP ≥100 mmHg but were not diagnosed or treated 34

Supplementary Table 11. Trends in SBP, DBP and raised BP prevalence by age group 35

Supplementary Table 12. Model fitting and comparisons of linear, quadratic, cubic, and cubic spline model 37

Supplement 1. Details of sampling scheme

The sample of CCDRFS was obtained from China's national Disease Surveillance Point (DSP) system.[1] One DSP unit covers a rural county or an urban district. The DSPs were established during early1980s, using multi-stage stratified sampling with probability proportional to size (PPS) to ensure representativeness of the national population of mainland China. Following further expansion and sampling enhancement, from the 2013 survey the selected DSPs were also representative at provincial level. CCDRFS is planned and administrated by the National Center for Chronic and Non-communicable Disease Control and Prevention (NCNCD) of the Chinese Center for Disease Control and Prevention (CDC).

Disease Surveillance Point (DSP)

The DSP system was piloted in 1978 and fully established in early 1980s covering initially 71 sites, expanding to 145 in 1989 and 161 DSPs in 2004 (covering a population of 73 million), in order to accommodate the societal and economic development during this period. For each expansion, the population characteristics of the selected DSPs were compared to the census population to ensure its nationally representativeness.[2-4] In 2013, the Chinese government combined the DSP system with the national vital registration system to form an integrated national mortality surveillance system, which increased the DSP areas from 161 to 605. The DSPs system currently covers 324 million Chinese adults (24% of all Chinese population) in the mainland of China.

Sampling procedure

Embedded within the DSP system, the CCDRFS used stratified multi-stage cluster sampling to generate a nationally representative sample for each survey.

In the first stage, half of the DSPs were selected as primary sampling units (PSUs) using stratified sampling (in the surveys of 2004, 2013, 2015 and 2018), or all DSPs were selected as PSUs (in the surveys of 2007 and 2010). Since the 2013 survey, following the expansion of the DSP system, 298 DSPs were selected from all 605 DSPs to generate a sample representative of both the national and provincial population in the 31 provinces of mainland China. The sampling of DSPs was done centrally by the NCNCD.

Then, within each selected DSP, the following steps were followed:

1. Townships (rural) or subdistricts (urban) were selected in each sampled DSP with PPS sampling using population size as the auxiliary variable in the surveys before 2015, and with simple random sampling in the surveys of 2015 and 2018.
2. Within the selected townships and subdistricts, villages (rural) or residential areas (urban) were selected with PPS sampling with PPS sampling in the surveys before 2015, and with simple random sampling in the surveys of 2015 and 2018.
3. Each selected village or residential area was divided into groups of about 50 households, based on existing villager/resident groups in the village or residential area. One group was selected with simple random sampling.
4. Within each selected household, in the 2004 survey, one eligible adult whose day of birth was closest to the 21st was invited. In the surveys of 2007, 2010, and 2013, the Kish Grid method was used to select one eligible adult. In the 2015 and 2018 surveys, all eligible adults in the household were invited, as a nutrition component was introduced in these surveys and all family members needed to be assessed to accurately calculate the nutrition intake.

   Eligibility criteria of the surveys include:

a. Aged 18 years or older;
b. Having lived in the address for more than 6 months in the past 12 months;
c. Not pregnant;
d. Not having a serious health condition or illness that prevents from participating, including intellectual disability or language disorder.

5. In a household, if the selected eligible adults refused to participate or were not reachable during the field survey period, he/she was considered non-response. In each round, response rate was > 90%.

For surveys between 2004 and 2013, the sampling within selected DSP, as described above, was conducted by the NCNCD centrally; since the 2015 survey, the sampling was performed by the provincial CDCs and the quality control was performed by the NCNCD. Summary sampling scheme of CCDRFS surveys were displayed in Supplementary fig 1.

Sample size calculation

Sample size of each round of CCDRFS was calculated using N = $deff\,\frac{u^2 p(1-p)}{d^2}$

where u = 1.96 (corresponding to 95% confidence level), *deff* is design efficiency (1.5 to 3.5), r is relative error (20%), and $d = r \times p$. p is the prevalence of factor studied for calculation. By using diabetes prevalence from previous national surveys (7-10%), the table below shows the calculated target sample sizes in each survey.

Survey	2004	2007	2010	2013	2015	2018
Target sample size	33,180	52,020	96,870	181,000	182,376	182,376

Sample weights

Across all CCDRFS surveys, we developed sample weights to account for multi-stage sampling design, survey non-response, and post-stratification. For an individual in the sample, his/her sample weight was computed as the product of the following three type of weights.

1. Base weights for multi-stage design (Wdesign)

   Wdesign = Wd1 × Wd2 × Wd3 × Wd4 × Wd5 × Wd6

   - Wd1 is the reciprocal of selection probability of the sampled DSP in the PSU stratum where the individual was from;
   - Wd2 is the reciprocal of selection probability of the sampled township (rural) or subdistrict (urban) in the PSU where the individual belonged;
   - Wd3 is the reciprocal of selection probability of the sampled village (rural) or residential area (urban) in the township or subdistrict where the individual was from;
   - Wd4 is the total number of groups in the village or residential area where the individual was from;
   - Wd5 is the total number of households in the group where the individual belonged divided by the number of selected households in the group where the individual was from.
   - Wd6 is the total number of eligible adults in the household where the individual was from (2013 or earlier) or 1 (since 2015).

2. Non-response weights (Wnr)

   For the surveys from 2004 to 2013, adjustment only considered non-response at household level as only an individual was selected in each household, and the Wnr were computed as the number of eligible households in the villager/resident group where the individual was from

divided by the number of participating households in the same villager/resident group. In the surveys of 2015 and 2018, Wnr were the product of two components: one was the non-response weights at household level which were computed as same as those of the previous surveys; the other was computed as the number of eligible adults in the household where the individual belonged divided by the number of respondents.

3. Post-stratification weights (Wps)

Stratifications included: province (31 levels), urban or rural (2 levels), gender (2 levels), age group (10 levels: 18-24, 25-29, 30-34, 35-39, 40-44, 45-49, 50-54, 55-59, 60-64, 65-69). The 2010 census population was also stratified in the same way. In kth stratum, the post-stratification weights (Wps,k) are:

$$\mathbf{Wps, k} = \frac{\textbf{Population in the } \mathbf{k^{th}} \textbf{ stratum of the 2010 census population}}{\textbf{Sum of Wdesign} \times \textbf{Wnr for all individuals in the } \mathbf{k^{th}} \textbf{ stratum}}$$

References

1. Yang G, Hu J, Rao KQ, et al. Mortality registration and surveillance in China: History, current situation and challenges [J]. Popul Health Metr, 2005, 3(1): 3.
2. Yang G. [Selection of DSP points in second stage and their presentation] [J]. Zhonghua Liu Xing Bing Xue Za Zhi, 1992, 13(4): 197-201.
3. Zhou MG, Jiang Y, Huang ZJ, et al. Adjustment and representativeness evaluation of national disease surveillance points system [J]. Dis Surveill, 2010, 25(3): 239-244.
4. Liu S, Wu X, Lopez AD, et al. An integrated national mortality surveillance system for death registration and mortality surveillance, China [J]. Bull World Health Organ, 2016, 94(1): 46-57.

Supplement 2. Further details on statistical analyses

1. Age-adjusted estimates for subgroups

For hypertension prevalence, awareness, treatment, control, treated control, and BP, subgroup analyses were also performed separately by residence (urban/rural), education (no education, primary school, secondary school, high school or above), geographical region (north, northeast, east, central, south, southwest, or northwest), BMI (underweight: BMI <18.5 kg/m^2, normal BMI: 18.5≤ BMI <25 kg/m^2, overweight: 25≤ BMI <30 kg/m^2, obese: BMI ≥30 kg/m^2), and central obesity (yes: waist circumference ≥90 cm for men or ≥85 cm for women; no: waist circumference <90 cm for men or <85 cm for women) among men and women. We produced age-adjusted estimates for each subgroup, so that direct comparisons could be made across sub-population and years independent on the age distribution of each group.

The age-adjusted estimates were calculated by design-based multivariable logistic regression for prevalence or linear regression for BP means, both of which contained categorical variables of survey year (2004/2007/2010/2013/2015/2018), sex, the subgroup variable, and their 2-way and 3-way interaction terms, including the continuous age as a covariate. Above analyses were conducted using *surveylogistic*[1] or *surveyreg*[2] procedure of SAS system with "lsmeans" statement, which obtained age-adjusted estimates (least squared means) and the 95% CIs. Specifically, we included all two-way and three-way interactions because the interactions terms were all significant in the model selection process.

The age-adjusted estimates or the LS-means are *predicted with linear models*. For ease of understanding how LS-means were obtained, we here demonstrate a simplified example: If we make comparisons of the trends in hypertension prevalence between men and women, the LS-means (prevalence in logit space) could be attained through the following model:

$$logit(P_{ij}) = \mu + \beta \cdot age + sex_i + year_j + \gamma_{ij}$$

Where *i*=1, 2, representing men and women, respectively; *j*=1, 2, 3, 4, 5, 6, representing the survey year of 2004, 2007, 2010, 2013, 2015, and 2018, respectively; P_{ij} is the prevalence of subpopulation indexed by (i, j); μ is the intercept; β is the coefficient of covariate age; sex_i and $year_j$ is the main effect of categorical variable sex and year; $\boldsymbol{\gamma_{ij}}$ **is the two-way interaction of sex and year**.

The age-adjusted population marginal means (in logit space) of the i th row or j th column are defined as the arithmetic means of population group means for groups of the i th row or j th column, while holding the age at a constant (often the sample mean). The age-adjusted means of the i th row and j th column can also be estimated with the same model. Let **φ** be the vector of parameters for the population [$\mu, \beta, sex_1, sex_2, year_1, \cdots, year_6,\ \gamma_{11}, \gamma_{12}, \cdots, \gamma_{25}, \gamma_{26}$]'. Then the age-adjusted population marginal means can be expressed as a linear combination of the population parameters **Lφ.** For example, the age-adjusted mean for both genders in 2004 can be represented as **Lφ=** $\mu + \beta \cdot age + 0.5 \cdot sex_1 + 0.5 \cdot sex_2 + year_1 + 0.5 \cdot \gamma_{11} + 0.5 \cdot \gamma_{21}$ with **L**=[1 age 0.5 0.5 1 0 0 0 0 0 0.5 0.5 0 0 0 0 0 0 0 0 0 0]; the age-adjusted mean for men in 2004 can be represented as **Lφ=** $\mu + \beta \cdot age + sex_1 + year_1 + \gamma_{11}$ with **L**=[1 age 1 0 1 0 0 0 0 0 1 0 0 0 0 0 0 0 0 0 0 0]. The age-adjusted prevalence can be readily calculated with the inverse logit function.

The above model could be easily expanded to models with higher order interactions while including more factors of interest and all age-adjusted estimates could be estimated in a similar way. Take the present study, the age-adjusted estimates were estimated through the following model with two-way and three-way interactions:

$$logit(P_{ij}) = \mu + \beta \cdot age + sex_i + year_j + f_k + \gamma_{ij} + \delta_{ik} + \theta_{jk} + \omega_{ijk}$$

Where f_k is the main effect of study factor of interest such as urban/rural, region, education, BMI, and central obesity; δ_{ik} is the two-way interaction of sex and the study factor; θ_{jk} is the two-way interaction of year and the study factor; ω_{ijk} is the three-way interaction of year, sex, and the study factor.

2. Trend analysis

We conducted preliminary analyses to assess whether trends in prevalence, awareness, treatment, and control rates had been nonlinear. The linear regression model was fitted by including year as the independent variable. The quadratic model was ordinary linear regression including year and quadratic term of year. The cubic model was ordinary linear regression including year, quadratic term of year, and cubic term of year. The spline model was generalised additive model containing year and penalised cubic regression spline term of year by applying 3 basis functions with two evenly spaced knots, using "gam" function from mgcv package in R. Adjusted R^2 was chosen to assess how well these models fitted the data. Then we conducted ANOVA F-test to compare the linear model with quadratic and cubic model. And we used Wald test for equality to zero of cubic spline terms in spline model.[3]

We then calculated annual change over the study period, as well as before and after 2010, when multiple national public health programmes were implemented. Absolute annual change in prevalence, awareness, treatment, and control levels from 2004 to 2010, from 2010 to 2018, and 2004 to 2018, overall and in groups were calculated as follows.

$$\text{annual change} = \frac{(\text{level}_{t2} - \text{level}_{t1})}{(t_2 - t_1)}$$

95% confidence interval for the annual change in rates is calculated using the Welch's t-test. The statistical significance of the difference in the annual changes between 2004-2010 and 2010-2018 in prevalence, awareness, treatment, control rates is also tested using the Welch's t-test.

We performed all analyses using procedures for survey data analysis, and estimated sampling error using Taylor series linearization with finite population correction.

References

1. SAS Institute Inc. SAS/STAT® 14.2 User's Guide. The SURVEYLOGISTIC Procedure; 2016.
2. SAS Institute Inc. SAS/STAT® 14.2 User's Guide. The SURVEYREG Procedure; 2016.
3. Wood SN. On p-values for smooth components of an extended generalized additive model. Biometrika 2012;100(1):221-28. doi: 10.1093/biomet/ass048 %J Biometrika

Supplement 3. Further details on blood pressure monitors

In each round of the CCDRFS survey, all respondents had their blood pressure (BP) measured three times successively with one-minute interval between measurements, using mercury sphygmomanometers in 2004, and electronic BP monitors from 2007 (HEM-770A in 2007, HEM-7071 in 2010, and HBP-1300 since 2013, Omron Company, Dalian, China).

According to the 2005 Chinese Guidelines for the Management of Hypertension, electronic BP monitors validated by British and Irish Hypertension Society (BHS) and the Association for the Advancement of Medical Instrumentation (AAMI) can be used in clinical practice. Mercury sphygmomanometer has been increasingly considered inappropriate for large-scale population survey due to listening fatigue during repetitive measurements and the general phase-out for medical use due to its environmental hazard. Since survey 2007, therefore, we adopted electronic BP monitors as measurement devices. Three models, HEM-770A, HEM-7071 and HBP-1300 (Omron Company, Dalian, China), were used in our study, and all of them had been validated and were approved by European Society of Hypertension (ESH), BHS and AAMI[1 2]. Before conducting each survey of CCDRFS, all electronic BP monitors were sent to Omron Company for calibration, and then half of them were randomly selected for further inspection by Beijing Bureau of Quality and Technical Supervision. Only those which have passed the inspection were used.

References

1. Liu X, Yu G, Zhang P. Accuracy Evaluation of OMRON HEM-770A Electronic Sphygmomanometer [J]. Chinese General Practice, 2011, 14: 1796-1797.
2. http://www.dableducational.org/sphygmomanometers/devices_1_clinical.html#ClinTable

Supplement 4. Mean BP and raised BP prevalence

Overall, mean SBP and mean DBP increased from 121.8 (120.9-122.8) mmHg and 76.8 (76.0-77.5) mmHg in 2004 to 128.7 (127.7-129.7) mmHg and 80.1 (79.6-80.6) mmHg in 2010 (both $P<0.001$), then declined to 126.4 (125.7-127.1) mmHg and 77.0 (76.6-77.5) mmHg in 2018 (both $P<0.001$). Similarly, raised BP prevalence increased from 19.3% (17.7-21.0) in 2004 to 27.9% (26.2-29.6) in 2010 ($P<0.001$) then to 21.7% (20.3-23.1) in 2018 ($P<0.001$).

The temporal trends of SBP, DBP and raised BP prevalence were broadly consistent with hypertension prevalence in different age groups among men and women (see supplementary table 11).

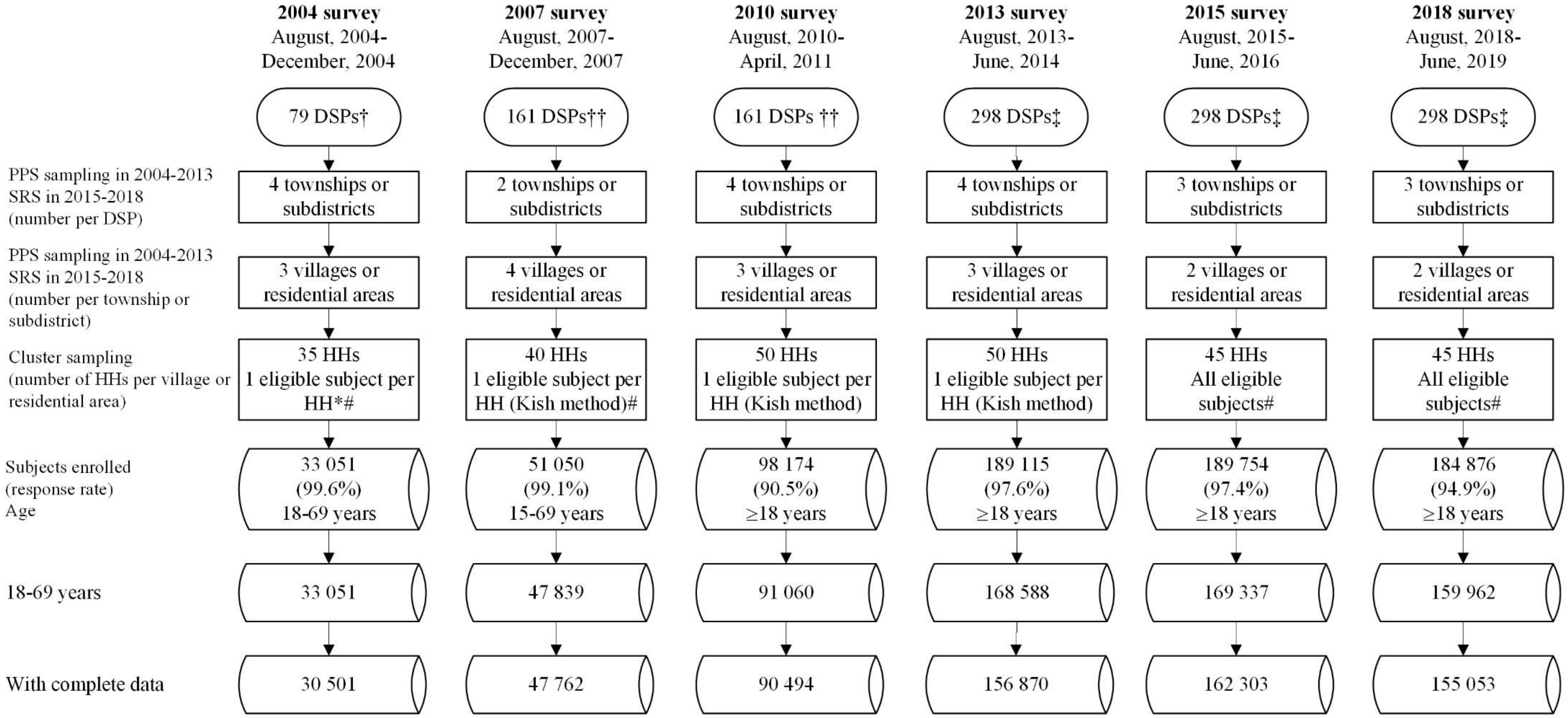

PPS: probability proportional to size; SRS: systematic random sampling; HH: household; DSP: Disease Surveillance Point.
Biochemical data were available from 2010. †Half of all DSPs (161) were selected using stratified sampling. ††All DSPs were selected. ‡Half of all DSPs (605) were selected using stratified sampling. *The subject whose day of birth was closest to the 21st was invited. #Eligiblity criteria include: a) aged 18 years or older; b) having lived in the address for more than 6 months in the past 12 months; c) not pregnant; d) not having a serious health condition or illness that prevents from participating, including intellectual disability or language disorder.

Supplementary Fig 1. Sampling scheme of CCDRFS surveys

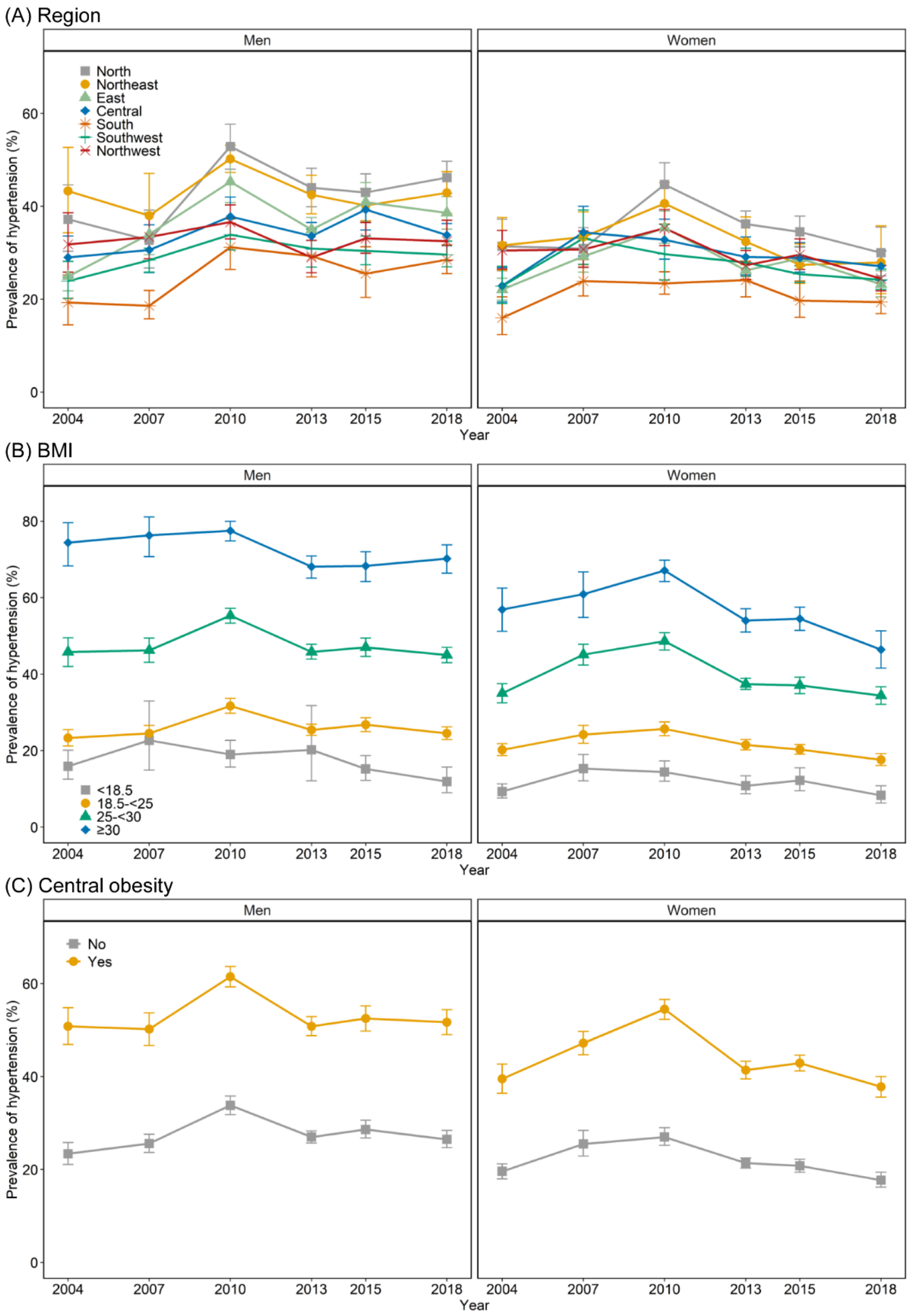

Supplementary Fig 2. Trends in adjusted hypertension prevalence by region, BMI and central obesity status among men and women from 2004 to 2018. (A) Hypertension prevalence were estimated using multivariable logistic regression containing age, survey year, sex, region, and 2-way and 3-way interaction terms of survey year, sex and region. (B) Hypertension prevalence were estimated using multivariable logistic regression containing age, survey year, sex, BMI, and 2-way and 3-way interaction terms of survey year, sex and BMI. (C) Hypertension prevalence were estimated using multivariable logistic regression containing age, survey year, sex, central obesity, and 2-way and 3-way interaction terms of survey year, sex and central obesity. Error bars indicate 95% confidence intervals with consideration of complex sample design.

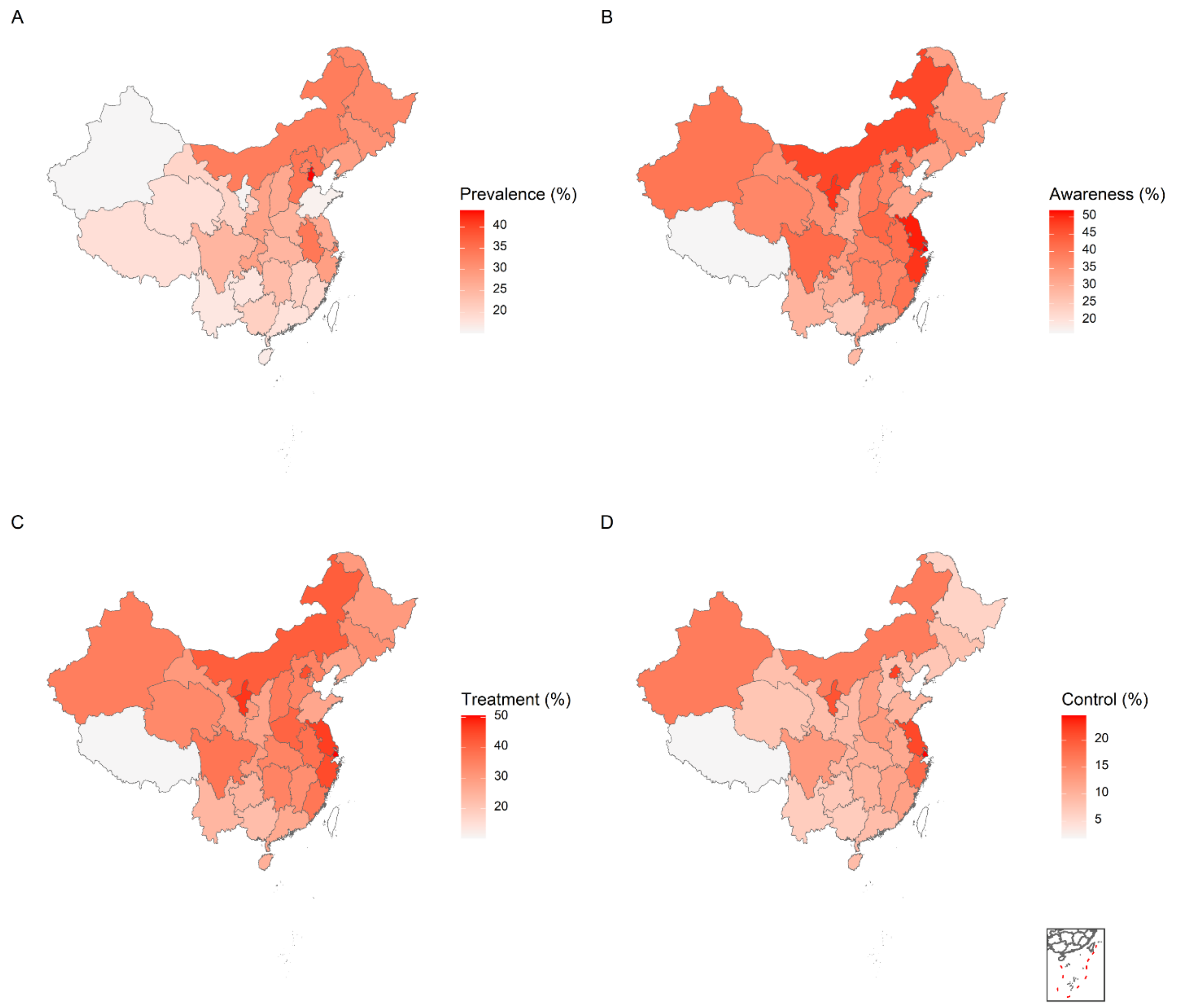

Supplementary Fig 3. The geographic variation in hypertension (A) prevalence, (B) awareness, (C) treatment and (D) control in China in 2018.

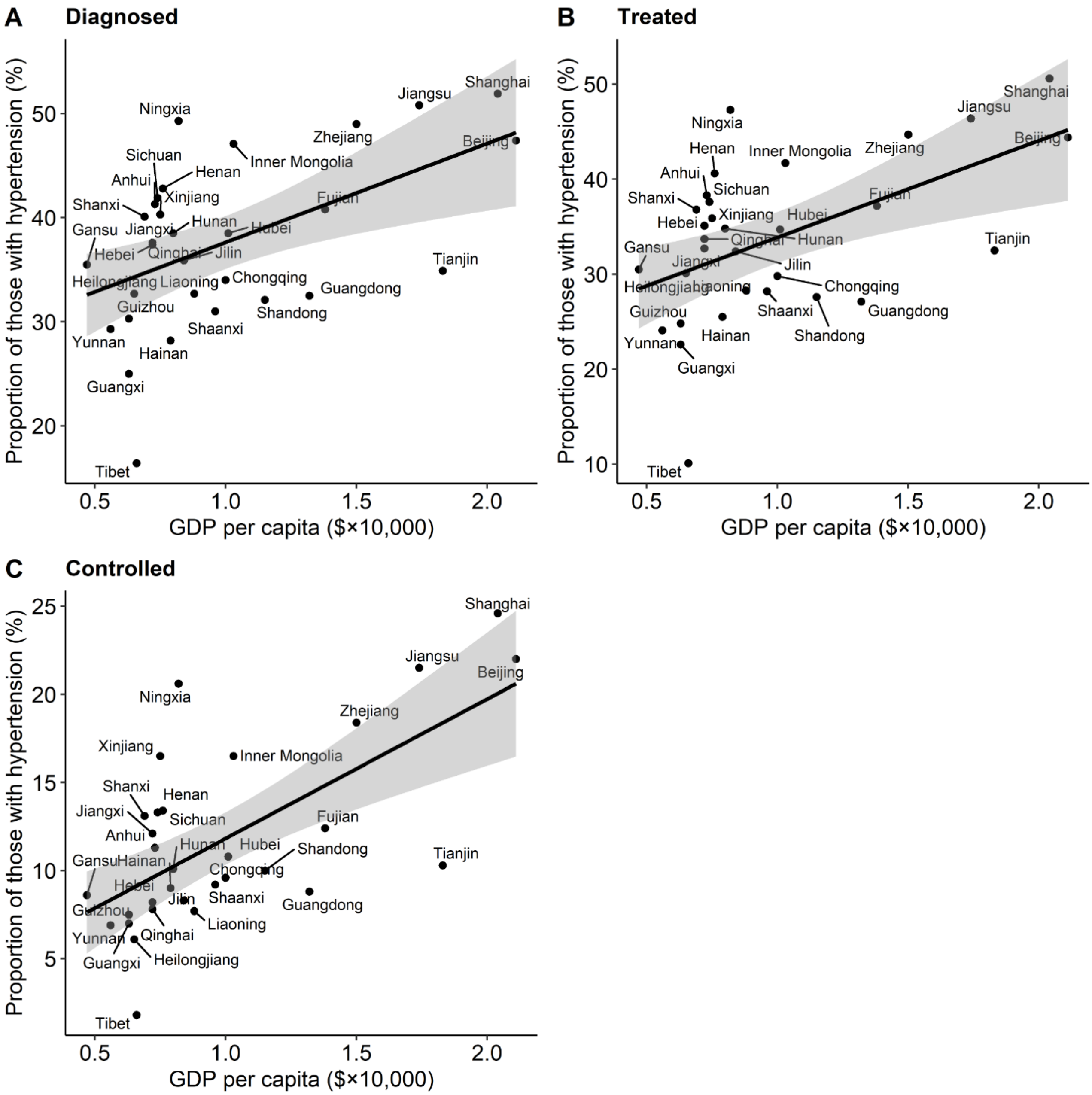

Supplementary Fig 4. Hypertension care cascade indicators by GDP per capita in different provinces of China in 2018.

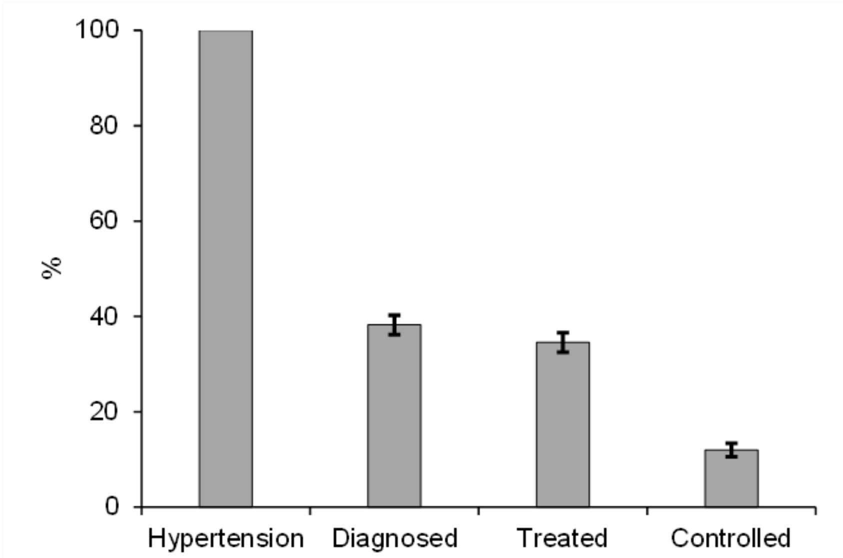

Supplementary Fig 5. The hypertension care cascade in China in 2018.

A Diagnosed (%)

Age	Men: No Education	Men: Primary school	Men: Secondary school	Men: High school or above	Women: No Education	Women: Primary school	Women: Secondary school	Women: High school or above
18-29	56.2	17.3	11.5	8.5	56.8	14.1	27.8	3.9
30-39	22.7	25.3	15	23.3	18.5	34.8	27.7	25.9
40-49	25.3	28.9	31	32.6	32.4	35.2	38	37.1
50-59	37.7	36.3	48.8	51.4	48.9	47.3	51	54.2
60-69	43.9	46.1	49.1	56.5	55.3	53.5	55.6	56.4

B Treated (%)

Age	Men: No Education	Men: Primary school	Men: Secondary school	Men: High school or above	Women: No Education	Women: Primary school	Women: Secondary school	Women: High school or above
18-29	46.1	17.3	9.9	5.3	55.8	2.8	26.6	3.1
30-39	22.1	14.2	12.8	19.8	13.6	29	24.8	24.1
40-49	20.3	23.7	27.3	28	28.2	31.4	35.7	34.3
50-59	32.8	32.6	43.6	47	44.8	44.5	48.2	51.3
60-69	38.2	41.2	45.8	53.3	51.4	50.7	53.8	55.7

C Controlled (%)

Age	Men: No Education	Men: Primary school	Men: Secondary school	Men: High school or above	Women: No Education	Women: Primary school	Women: Secondary school	Women: High school or above
18-29	0	0	0.1	3.7	4.8	2.8	6.5	1.7
30-39	6.7	6.9	6.8	8.3	4.8	6.3	8.1	15.5
40-49	4.3	6.6	11.1	9.1	6.7	8.5	10.9	16.4
50-59	8.7	10.2	12.9	16.6	12.8	13.3	20.5	22.5
60-69	11.1	12.8	15.9	21.7	15	18.3	20.9	25.2

Supplementary Fig 6. Proportion of participants with hypertension reaching each cascade step, stratified by sex, age group, and education in 2018.

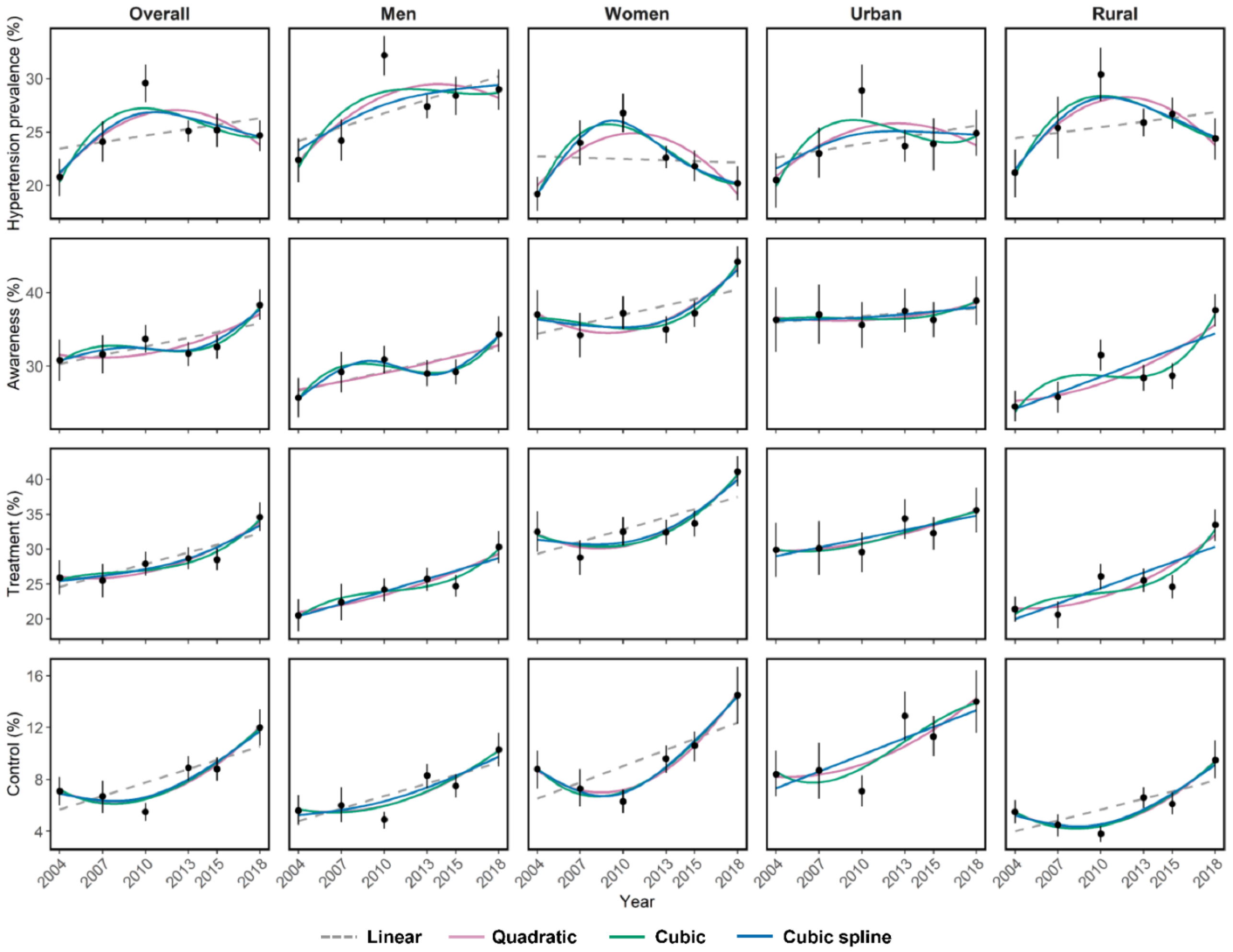

Supplementary Fig 7. Linear and nonlinear trends of hypertension prevalence, awareness, treatment, and control with survey year.

Supplementary Table 1. Demographics characteristic of CCDRFS surveys[a]

	2004 (n=30501)	2007 (n=47353)	2010 (n=90491)	2013 (n=156836)	2015 (n=162293)	2018 (n=155049)
**Sex**						
Men	13430 (44.0)	22408 (47.3)	41238 (45.6)	66370 (42.3)	74732 (46.0)	67160 (43.3)
Women	17071 (56.0)	24945 (52.7)	49253 (54.4)	90466 (57.7)	87561 (54.0)	87889 (56.7)
**Age, years**						
18-29	3583 (11.7)	6219 (13.1)	14554 (16.1)	14011 (8.9)	15881 (9.8)	9386 (6.1)
30-39	7888 (25.9)	11435 (24.1)	17796 (19.7)	22554 (14.4)	21781 (13.4)	17688 (11.4)
40-49	8323 (27.3)	12038 (25.4)	24677 (27.3)	43077 (27.5)	40083 (24.7)	32263 (20.8)
50-59	6720 (22.0)	10975 (23.2)	20812 (23.0)	43831 (27.9)	44320 (27.3)	47043 (30.3)
60-69	3987 (13.1)	6686 (14.1)	12652 (14.0)	33363 (21.3)	40228 (24.8)	48669 (31.4)
**Residence**						
Urban	11659 (38.2)	18359 (38.8)	35705 (39.5)	65061 (41.5)	68812 (42.4)	70716 (45.6)
Rural	18842 (61.8)	28994 (61.2)	54786 (60.5)	91775 (58.5)	93481 (57.6)	84333 (54.4)
**BMI,** kg/m^2						
<18.5	1983 (6.5)	2234 (4.7)	3371 (3.7)	4968 (3.2)	5410 (3.3)	4041 (2.6)
18.5-	20878 (68.5)	32255 (68.1)	55340 (61.2)	88929 (56.7)	93592 (57.7)	83978 (54.2)
25-	6556 (21.5)	11242 (23.7)	26647 (29.4)	52077 (33.2)	52833 (32.6)	55363 (35.7)
30-	1084 (3.6)	1622 (3.4)	5133 (5.7)	10862 (6.9)	10458 (6.4)	11667 (7.5)
**Central obesity [b]**						
No	24079 (78.9)	37213 (78.6)	66498 (73.5)	100073 (63.8)	109632 (67.6)	93206 (60.1)
Yes	6422 (21.1)	10140 (21.4)	23993 (26.5)	56763 (36.2)	52661 (32.4)	61843 (39.9)
**Education**						
No Education	5590 (18.3)	11814 (24.9)	19554 (21.6)	39435 (25.1)	42299 (26.1)	40928 (26.4)
Primary school	8844 (29.0)	9302 (19.6)	17483 (19.3)	31295 (20.0)	32565 (20.1)	30273 (19.5)
Secondary school	9979 (32.7)	15318 (32.3)	30162 (33.3)	52077 (33.2)	52572 (32.4)	50280 (32.4)
High school or above	6088 (20.0)	10919 (23.1)	23292 (25.7)	34029 (21.7)	34857 (21.5)	33568 (21.6)
**Region**						
North	4159 (13.6)	6730 (14.2)	12876 (14.2)	23011 (14.7)	23275 (14.3)	22411 (14.5)

Northeast	3538 (11.6)	5069 (10.7)	10110 (11.2)	15275 (9.7)	15542 (9.6)	14881 (9.6)
East	7205 (23.6)	11823 (25.0)	21789 (24.1)	40742 (26.0)	41145 (25.4)	38593 (24.9)
Central	3341 (11.0)	6224 (13.1)	12211 (13.5)	19459 (12.4)	20002 (12.3)	19377 (12.5)
South	2753 (9.0)	4157 (8.8)	7693 (8.5)	15353 (9.8)	16089 (9.9)	15724 (10.1)
Southwest	5059 (16.6)	7484 (15.8)	14712 (16.3)	22639 (14.4)	24796 (15.3)	23316 (15.0)
Northwest	4446 (14.6)	5866 (12.4)	11100 (12.3)	20357 (13.0)	21444 (13.2)	20747 (13.4)

Abbreviation: CCDRFS, China Chronic Disease and Risk Factors Surveillance.

[a] Numbers and unweighted proportions (%).

[b] Central obesity was defined as having waist circumference ≥90 cm for men and ≥85 cm for women.

Supplementary Table 2. Trends in hypertension prevalence, awareness, treatment, and control rates in urban and rural areas (%)[a]

Sex	Prevalence	Residence	2004	2007	2010	2013	2015	2018
Men	Prevalence	Urban	30.3 (26.4 to 34.5)	31.3 (27.9 to 35.0)	43.3 (40.1 to 46.5)	36.2 (34.0 to 38.6)	36.4 (33.2 to 39.6)	40.9 (38.5 to 43.3)
		Rural	28.1 (24.5 to 32.0)	30.9 (28.2 to 33.6)	40.3 (37.4 to 43.3)	34.5 (32.7 to 36.4)	37.1 (34.5 to 39.8)	33.5 (31.6 to 35.5)
	Awareness	Urban	35.3 (30.7 to 40.2)	39.7 (35.1 to 44.5)	39.5 (36.7 to 42.5)	41.6 (38.1 to 45.2)	38.3 (35.5 to 41.3)	43.0 (38.7 to 47.4)
		Rural	22.7 (20.4 to 25.2)	27.2 (24.1 to 30.4)	33.7 (31.0 to 36.5)	30.6 (28.3 to 33.0)	29.5 (27.3 to 31.7)	38.1 (35.3 to 40.9)
	Treatment	Urban	27.8 (23.7 to 32.3)	31.6 (27.4 to 36.2)	31.4 (28.7 to 34.2)	38.0 (34.6 to 41.6)	33.4 (30.6 to 36.4)	39.2 (34.9 to 43.6)
		Rural	18.7 (16.7 to 20.9)	19.9 (16.8 to 23.4)	26.7 (24.3 to 29.2)	26.8 (24.6 to 29.2)	24.4 (22.4 to 26.5)	32.9 (30.2 to 35.7)
	Control	Urban	7.4 (5.5 to 9.7)	8.8 (6.5 to 11.8)	7.0 (5.9 to 8.3)	13.8 (11.6 to 16.4)	10.7 (9.0 to 12.6)	13.8 (11.4 to 16.5)
		Rural	5.1 (4.0 to 6.5)	4.5 (3.4 to 6.0)	4.1 (3.2 to 5.1)	7.2 (6.2 to 8.3)	6.1 (5.2 to 7.2)	9.6 (7.9 to 11.5)
Women	Prevalence	Urban	24.2 (21.4 to 27.1)	29.1 (26.6 to 31.6)	32.9 (30.3 to 35.7)	27.1 (25.2 to 29.0)	25.9 (23.8 to 28.1)	24.1 (21.4 to 26.9)
		Rural	25.7 (23.1 to 28.5)	33.0 (29.8 to 36.4)	36.8 (33.9 to 39.8)	29.5 (27.7 to 31.3)	29.8 (28.2 to 31.5)	27.2 (25.6 to 28.8)
	Awareness	Urban	45.2 (39.5 to 51.1)	42.8 (38.7 to 47.0)	43.3 (39.2 to 47.5)	46.5 (43.3 to 49.9)	43.2 (39.9 to 46.6)	49.0 (45.4 to 52.5)
		Rural	32.5 (29.5 to 35.7)	30.9 (27.2 to 34.9)	38.3 (35.7 to 41.1)	34.1 (31.8 to 36.4)	34.4 (32.4 to 36.5)	44.5 (42.1 to 46.9)
	Treatment	Urban	38.9 (33.8 to 44.2)	35.8 (31.9 to 39.9)	38.3 (34.5 to 42.3)	43.6 (40.5 to 46.8)	39.6 (36.4 to 42.9)	46.2 (42.7 to 49.9)
		Rural	29.5 (26.6 to 32.5)	26.2 (23.5 to 29.1)	33.3 (30.9 to 35.8)	31.6 (29.4 to 33.8)	30.6 (28.6 to 32.7)	40.8 (38.3 to 43.4)
	Control	Urban	11.0 (8.8 to 13.7)	10.1 (7.6 to 13.4)	9.2 (7.5 to 11.1)	15.7 (13.5 to 18.2)	14.4 (12.3 to 16.8)	19.2 (15.0 to 24.4)
		Rural	7.0 (5.6 to 8.7)	5.3 (4.3 to 6.4)	4.3 (3.6 to 5.2)	7.5 (6.5 to 8.6)	7.2 (6.2 to 8.5)	10.9 (9.3 to 12.7)

[a] The prevalence and rates were estimated using multivariable logistic regression containing age, survey year, sex, residence, and 2-way and 3-way interaction terms of survey year, sex and residence.

Supplementary Table 3. Trends in hypertension prevalence, awareness, treatment, and control rates by education (%)[a]

Sex	Rate	Education	2004	2007	2010	2013	2015	2018
Men	Prevalence	No Education	24.7 (21.4 to 28.4)	25.5 (23.3 to 27.7)	34.7 (32.2 to 37.2)	31.4 (28.7 to 34.3)	33.1 (31.0 to 35.3)	31.8 (29.5 to 34.2)
		Primary school	26.9 (23.3 to 30.9)	27.5 (25.0 to 30.2)	38.1 (36.0 to 40.4)	31.1 (29.3 to 33.1)	34.2 (32.0 to 36.6)	33.3 (31.1 to 35.6)
		Secondary school	31.8 (28.0 to 35.8)	34.8 (31.7 to 38.0)	44.0 (41.3 to 46.7)	37.1 (35.3 to 38.9)	39.7 (37.1 to 42.4)	39.1 (36.9 to 41.5)
		High school or above	29.8 (26.6 to 33.1)	31.8 (28.1 to 35.7)	43.5 (40.6 to 46.4)	36.3 (34.3 to 38.5)	35.5 (32.6 to 38.5)	38.6 (36.0 to 41.3)
	Awareness	No Education	18.1 (14.7 to 22.1)	21.0 (17.6 to 24.8)	28.5 (25.6 to 31.5)	25.8 (23.6 to 28.1)	28.8 (26.0 to 31.7)	35.9 (31.6 to 40.4)
		Primary school	24.5 (21.1 to 28.3)	27.4 (23.2 to 32.1)	31.8 (29.1 to 34.7)	29.8 (27.3 to 32.4)	29.0 (26.3 to 31.9)	37.2 (34.4 to 40.1)
		Secondary school	31.9 (28.1 to 36.0)	35.8 (30.8 to 41.1)	36.3 (33.7 to 39.0)	36.1 (33.3 to 38.9)	35.4 (32.8 to 38.2)	42.1 (37.7 to 46.6)
		High school or above	39.5 (34.9 to 44.4)	46.7 (42.8 to 50.7)	46.8 (43.9 to 49.6)	44.1 (40.5 to 47.7)	40.2 (36.6 to 43.9)	44.9 (40.8 to 49.1)
	Treatment	No Education	15.0 (11.9 to 18.7)	17.5 (14.3 to 21.1)	22.7 (20.3 to 25.3)	22.5 (20.4 to 24.8)	24.1 (21.7 to 26.8)	30.2 (26.1 to 34.7)
		Primary school	20.8 (17.5 to 24.6)	21.8 (18.4 to 25.7)	25.5 (23.2 to 28.0)	26.4 (24.1 to 28.8)	24.7 (22.5 to 27.2)	31.7 (29.1 to 34.5)
		Secondary school	24.7 (21.5 to 28.3)	26.6 (22.2 to 31.5)	29.0 (26.5 to 31.7)	32.2 (29.4 to 35.1)	30.0 (27.4 to 32.8)	38.1 (33.8 to 42.6)
		High school or above	31.1 (27.3 to 35.1)	36.6 (32.5 to 40.9)	36.8 (34.0 to 39.6)	40.0 (36.6 to 43.6)	34.8 (31.5 to 38.3)	40.6 (37.0 to 44.3)
	Control	No Education	3.9 (2.1 to 7.1)	3.5 (2.4 to 5.2)	3.7 (2.9 to 4.8)	6.4 (5.1 to 7.9)	6.6 (5.3 to 8.1)	7.7 (6.5 to 9.1)
		Primary school	6.4 (4.0 to 10.1)	5.0 (3.5 to 7.3)	4.4 (3.5 to 5.6)	7.1 (5.9 to 8.4)	7.1 (5.8 to 8.5)	9.4 (7.9 to 11.1)
		Secondary school	6.0 (4.6 to 7.9)	6.8 (4.4 to 10.3)	5.2 (4.2 to 6.4)	9.5 (8.2 to 11.1)	8.3 (7.0 to 9.7)	12.6 (10.5 to 15.1)
		High school or above	8.3 (6.3 to 10.8)	11.2 (8.6 to 14.5)	8.7 (7.4 to 10.3)	15.0 (12.6 to 17.8)	11.5 (9.4 to 14.0)	14.8 (12.0 to 18.1)
Women	Prevalence	No Education	27.5 (24.9 to 30.2)	35.4 (31.6 to 39.3)	38.8 (36.0 to 41.6)	30.5 (28.9 to 32.1)	33.7 (31.8 to 35.7)	31.2 (29.6 to 32.8)
		Primary school	26.5 (23.6 to 29.6)	32.9 (30.4 to 35.4)	37.2 (34.6 to 39.9)	29.5 (27.9 to 31.1)	31.2 (29.5 to 32.9)	29.4 (27.8 to 31.2)
		Secondary school	24.8 (22.2 to 27.5)	29.5 (27.2 to 31.9)	36.0 (33.6 to 38.6)	29.1 (27.2 to 31.0)	27.6 (25.6 to 29.6)	26.8 (24.4 to 29.3)
		High school or above	19.2 (17.1 to 21.5)	25.2 (22.2 to 28.4)	27.4 (25.1 to 29.8)	24.2 (22.3 to 26.3)	20.0 (18.1 to 22.0)	18.3 (15.8 to 21.1)
	Awareness	No Education	30.7 (26.0 to 35.9)	30.6 (26.8 to 34.6)	35.9 (32.7 to 39.1)	31.4 (29.1 to 33.7)	35.0 (32.9 to 37.3)	44.6 (42.0 to 47.1)
		Primary school	39.0 (35.2 to 42.9)	40.1 (36.2 to 44.1)	39.1 (36.6 to 41.7)	37.9 (35.3 to 40.6)	38.4 (34.2 to 42.8)	45.7 (42.9 to 48.7)
		Secondary school	46.6 (41.9 to 51.4)	41.9 (36.8 to 47.1)	44.2 (41.1 to 47.3)	41.7 (39.0 to 44.4)	42.1 (39.4 to 44.9)	49.6 (45.7 to 53.4)
		High school or above	49.5 (43.1 to 55.9)	43.8 (40.2 to 47.4)	49.6 (45.5 to 53.7)	53.8 (50.0 to 57.5)	43.1 (39.6 to 46.7)	47.8 (41.2 to 54.5)
	Treatment	No Education	26.7 (22.4 to 31.4)	24.6 (22.0 to 27.4)	31.1 (28.2 to 34.2)	28.5 (26.4 to 30.7)	30.7 (28.7 to 32.9)	40.1 (37.4 to 42.8)

Sex	Rate	Education	2004	2007	2010	2013	2015	2018
		Primary school	34.3 (30.9 to 37.8)	33.3 (29.6 to 37.3)	34.2 (31.9 to 36.6)	35.6 (33.0 to 38.1)	35.1 (30.8 to 39.6)	41.8 (39.1 to 44.6)
		Secondary school	41.4 (36.7 to 46.1)	36.0 (31.1 to 41.1)	38.9 (36.0 to 41.8)	39.3 (36.7 to 42.0)	38.4 (35.7 to 41.3)	47.2 (43.2 to 51.2)
		High school or above	44.0 (38.0 to 50.2)	39.4 (35.8 to 43.1)	44.2 (40.3 to 48.2)	50.9 (47.2 to 54.6)	40.0 (36.9 to 43.2)	45.8 (39.0 to 52.8)
	Control	No Education	5.1 (3.8 to 6.8)	4.4 (3.6 to 5.3)	4.2 (3.5 to 5.1)	7.1 (6.2 to 8.2)	7.7 (6.5 to 9.1)	10.9 (9.6 to 12.3)
		Primary school	8.9 (6.9 to 11.5)	8.6 (6.6 to 11.0)	5.3 (4.3 to 6.4)	9.4 (8.0 to 11.1)	10.7 (7.8 to 14.4)	12.7 (11.0 to 14.7)
		Secondary school	12.9 (9.6 to 17.1)	9.1 (6.6 to 12.5)	8.3 (6.8 to 10.1)	11.8 (9.9 to 13.9)	11.8 (10.1 to 13.8)	17.3 (13.7 to 21.6)
		High school or above	14.5 (11.0 to 18.8)	14.7 (10.3 to 20.5)	12.8 (10.3 to 15.8)	18.9 (16.1 to 22.0)	17.1 (14.6 to 19.9)	21.2 (15.2 to 28.9)

[a] The prevalence and rates were estimated using multivariable logistic regression containing age, survey year, sex, education, and 2-way and 3-way interaction terms of survey year, sex and education.

Supplementary Table 4. Trends in standardised hypertension prevalence, awareness, treatment, and control rates by age group (%)[a]

Sex	Rate	Age	2004	2007	2010	2013	2015	2018
Men	Prevalence	18-29	7.2 (5.4 to 9.0)	9.2 (7.3 to 11.2)	13.2 (11.4 to 15.0)	12.5 (10.9 to 14.0)	10.5 (8.8 to 12.3)	13.3 (11.2 to 15.3)
		30-39	14.1 (11.9 to 16.3)	16.6 (14.8 to 18.4)	25.3 (22.9 to 27.6)	20.4 (18.8 to 22.0)	19.9 (17.5 to 22.3)	20.4 (18.4 to 22.3)
		40-49	25.2 (22.5 to 27.9)	26.4 (23.9 to 28.8)	35.9 (33.9 to 37.9)	29.4 (28.1 to 30.7)	32.4 (30.1 to 34.6)	31.8 (30.2 to 33.3)
		50-59	37.3 (34.6 to 39.9)	39.3 (36.6 to 42.0)	49.3 (47.4 to 51.2)	42.3 (40.8 to 43.8)	46.0 (44.3 to 47.6)	46.5 (44.3 to 48.8)
		60-69	50.2 (47.1 to 53.4)	52.1 (49.8 to 54.5)	61.9 (60.0 to 63.7)	52.7 (51.1 to 54.2)	57.4 (55.9 to 58.9)	55.3 (53.3 to 57.2)
	Awareness	18-29	8.4 (2.7 to 14.1)	6.5 (2.5 to 10.6)	9.9 (7.3 to 12.5)	9.4 (5.2 to 13.5)	5.8 (3.9 to 7.7)	11.4 (4.9 to 17.9)
		30-39	11.0 (7.8 to 14.2)	19.4 (15.6 to 23.1)	19.7 (17.1 to 22.3)	17.5 (14.9 to 20.1)	16.5 (13.5 to 19.4)	20.2 (16.9 to 23.4)
		40-49	23.6 (19.9 to 27.2)	25.8 (21.7 to 29.8)	29.2 (26.8 to 31.6)	28.4 (26.0 to 30.8)	26.6 (24.0 to 29.2)	30.8 (27.4 to 34.2)
		50-59	30.7 (26.9 to 34.6)	36.8 (32.7 to 40.9)	38.7 (36.1 to 41.2)	36.1 (33.9 to 38.4)	36.2 (33.9 to 38.5)	46.5 (42.4 to 50.7)
		60-69	37.9 (34.0 to 41.8)	41.5 (37.0 to 46.0)	45.0 (42.5 to 47.5)	42.3 (40.2 to 44.5)	44.6 (42.3 to 46.9)	48.7 (46.1 to 51.3)
	Treatment	18-29	5.1 (1.1 to 9.0)	2.2 (0.2 to 4.1)	5.5 (3.8 to 7.2)	8.8 (4.8 to 12.9)	3.1 (1.7 to 4.6)	8.7 (3.3 to 14.0)
		30-39	6.8 (4.4 to 9.1)	11.7 (8.8 to 14.6)	12.9 (10.6 to 15.2)	14.0 (11.8 to 16.3)	12.2 (9.9 to 14.4)	16.7 (13.4 to 19.9)
		40-49	17.4 (14.6 to 20.2)	19.5 (15.8 to 23.1)	21.9 (19.9 to 24.0)	24.4 (22.0 to 26.7)	21.8 (19.6 to 24.1)	26.5 (23.3 to 29.7)
		50-59	25.2 (21.7 to 28.7)	29.0 (25.2 to 32.9)	31.7 (29.3 to 34.2)	32.4 (30.2 to 34.6)	31.3 (29.0 to 33.6)	41.8 (37.9 to 45.7)
		60-69	32.7 (28.5 to 36.8)	34.9 (30.6 to 39.2)	38.3 (35.8 to 40.7)	38.6 (36.4 to 40.7)	40.1 (37.7 to 42.6)	44.5 (41.6 to 47.4)
	Control	18-29	0.8 (0.0 to 1.9)	1.6 (0.0 to 3.5)	1.7 (0.8 to 2.6)	3.0 (1.1 to 4.8)	1.4 (0.3 to 2.5)	2.2 (0.0 to 4.5)
		30-39	3.4 (1.7 to 5.2)	3.9 (1.9 to 5.9)	2.7 (2.0 to 3.5)	4.2 (2.9 to 5.5)	4.3 (2.9 to 5.7)	7.5 (5.2 to 9.9)
		40-49	5.3 (3.8 to 6.8)	5.0 (3.6 to 6.4)	4.6 (3.8 to 5.4)	8.2 (6.9 to 9.4)	6.1 (5.0 to 7.2)	9.2 (7.1 to 11.4)
		50-59	6.5 (4.6 to 8.4)	7.8 (5.7 to 10.0)	6.8 (5.6 to 8.0)	11.0 (9.5 to 12.4)	10.2 (8.8 to 11.6)	13.1 (11.5 to 14.7)
		60-69	8.2 (6.2 to 10.2)	8.6 (6.4 to 10.8)	6.5 (5.5 to 7.5)	11.7 (10.1 to 13.2)	11.1 (9.7 to 12.5)	15.3 (13.6 to 16.9)
Women	Prevalence	18-29	2.6 (1.8 to 3.5)	5.5 (4.1 to 6.8)	6.8 (5.5 to 8.1)	7.6 (6.0 to 9.2)	3.2 (2.5 to 3.9)	4.0 (2.9 to 5.1)
		30-39	7.8 (6.4 to 9.2)	11.0 (9.7 to 12.4)	14.2 (12.6 to 15.9)	10.9 (9.7 to 12.1)	8.7 (7.7 to 9.7)	8.4 (6.8 to 10.1)
		40-49	20.8 (18.9 to 22.8)	27.2 (24.8 to 29.7)	29.6 (27.7 to 31.5)	22.6 (21.5 to 23.7)	23.9 (22.5 to 25.3)	20.8 (19.4 to 22.2)
		50-59	39.1 (36.3 to 41.9)	45.0 (42.6 to 47.4)	50.0 (47.4 to 52.5)	41.8 (40.5 to 43.2)	43.9 (41.9 to 46.0)	40.9 (39.4 to 42.4)
		60-69	52.5 (49.9 to 55.1)	61.6 (57.8 to 65.4)	64.6 (62.7 to 66.5)	55.9 (54.5 to 57.3)	60.1 (58.3 to 61.9)	54.6 (53.0 to 56.3)
	Awareness	18-29	21.7 (6.0 to 37.3)	7.2 (3.2 to 11.2)	13.0 (8.3 to 17.7)	13.8 (9.2 to 18.4)	7.0 (2.9 to 11.1)	17.6 (6.3 to 28.8)

Sex	Rate	Age	2004	2007	2010	2013	2015	2018
		30-39	19.2 (14.6 to 23.8)	19.3 (15.3 to 23.3)	18.8 (15.8 to 21.7)	24.2 (20.0 to 28.3)	17.6 (14.2 to 21.0)	26.6 (21.3 to 31.8)
		40-49	28.6 (24.6 to 32.6)	30.2 (26.3 to 34.0)	33.0 (30.5 to 35.4)	30.4 (28.1 to 32.6)	28.7 (24.6 to 32.9)	36.1 (33.2 to 38.9)
		50-59	42.7 (38.3 to 47.2)	37.7 (32.5 to 42.8)	43.7 (41.0 to 46.5)	40.0 (38.1 to 42.0)	41.9 (39.8 to 44.0)	50.3 (47.4 to 53.1)
		60-69	45.3 (40.9 to 49.8)	46.5 (42.6 to 50.4)	49.1 (46.4 to 51.9)	45.0 (42.6 to 47.3)	49.7 (47.5 to 51.9)	55.2 (52.9 to 57.5)
	Treatment	18-29	21.7 (6.0 to 37.3)	5.8 (2.5 to 9.0)	10.1 (6.0 to 14.2)	12.2 (7.9 to 16.6)	6.3 (2.2 to 10.4)	14.9 (3.8 to 26.0)
		30-39	15.3 (11.3 to 19.3)	15.1 (10.9 to 19.3)	14.0 (11.6 to 16.5)	21.6 (17.7 to 25.5)	13.2 (10.4 to 16.1)	23.5 (18.1 to 28.9)
		40-49	24.6 (21.3 to 27.9)	23.6 (20.8 to 26.4)	27.4 (25.3 to 29.5)	27.6 (25.6 to 29.7)	25.3 (21.0 to 29.6)	32.9 (30.3 to 35.5)
		50-59	36.9 (32.9 to 41.0)	32.4 (28.0 to 36.8)	39.0 (36.4 to 41.5)	37.3 (35.4 to 39.2)	38.5 (36.5 to 40.5)	47.1 (44.1 to 50.1)
		60-69	41.3 (36.8 to 45.9)	40.7 (37.5 to 44.0)	45.2 (42.4 to 48.0)	42.5 (40.2 to 44.8)	46.0 (43.6 to 48.3)	52.3 (49.9 to 54.6)
	Control	18-29	10.7 (0.4 to 21.0)	2.5 (0.6 to 4.3)	5.1 (2.6 to 7.5)	5.0 (2.0 to 7.9)	3.7 (0.3 to 7.2)	3.8 (0.0 to 7.7)
		30-39	6.3 (3.4 to 9.1)	5.8 (3.2 to 8.3)	3.1 (2.1 to 4.1)	5.3 (3.5 to 7.1)	4.1 (2.6 to 5.7)	10.5 (4.3 to 16.6)
		40-49	6.5 (4.8 to 8.2)	5.8 (4.1 to 7.5)	5.5 (4.5 to 6.4)	8.7 (7.4 to 10.0)	9.1 (6.5 to 11.6)	10.7 (8.4 to 12.9)
		50-59	9.9 (7.5 to 12.3)	7.5 (4.9 to 10.0)	7.3 (6.0 to 8.6)	11.7 (10.4 to 12.9)	12.5 (11.0 to 14.1)	17.3 (14.3 to 20.3)
		60-69	10.0 (7.8 to 12.1)	10.5 (7.9 to 13.2)	8.0 (6.7 to 9.3)	11.2 (9.8 to 12.5)	12.6 (10.9 to 14.2)	18.0 (16.1 to 19.9)

[a] Results were weighted to the 2010 census population in China.

Supplementary Table 5. Trends in hypertension prevalence, awareness, treatment, and control rates by region (%)[a]

Sex	Rate	Region	2004	2007	2010	2013	2015	2018
Men	Prevalence	North	37.2 (30.3 to 44.6)	32.7 (26.7 to 39.2)	52.9 (48.0 to 57.7)	44.0 (39.9 to 48.2)	43.0 (39.0 to 47.0)	46.2 (42.8 to 49.7)
		Northeast	43.3 (34.3 to 52.7)	38.0 (29.6 to 47.1)	50.2 (47.3 to 53.2)	42.5 (38.4 to 46.7)	40.2 (36.9 to 43.6)	42.9 (38.5 to 47.5)
		East	24.9 (21.8 to 28.2)	34.0 (29.6 to 38.6)	45.3 (40.8 to 49.8)	35.0 (32.6 to 37.6)	40.9 (36.7 to 45.1)	38.6 (35.1 to 42.1)
		Central	29.0 (24.8 to 33.6)	30.6 (25.7 to 36.0)	37.8 (33.8 to 42.0)	33.6 (30.8 to 36.5)	39.3 (34.9 to 43.8)	33.8 (31.5 to 36.3)
		South	19.3 (14.5 to 25.2)	18.6 (15.8 to 21.9)	31.2 (26.4 to 36.4)	29.3 (24.8 to 34.4)	25.5 (20.4 to 31.3)	28.5 (25.5 to 31.7)
		Southwest	23.9 (20.2 to 28.1)	28.4 (25.8 to 31.0)	33.9 (30.6 to 37.3)	30.9 (26.9 to 35.1)	30.4 (27.4 to 33.6)	29.6 (27.0 to 32.5)
		Northwest	31.8 (25.8 to 38.6)	33.4 (28.7 to 38.4)	36.6 (33.0 to 40.3)	29.0 (25.7 to 32.7)	33.1 (29.9 to 36.5)	32.5 (28.3 to 37.0)
	Awareness	North	36.0 (27.6 to 45.3)	43.7 (37.2 to 50.4)	38.7 (33.1 to 44.6)	37.4 (33.1 to 41.8)	38.5 (35.3 to 41.9)	44.9 (35.8 to 54.3)
		Northeast	26.3 (22.0 to 31.1)	28.9 (21.8 to 37.3)	35.0 (29.6 to 40.8)	30.2 (26.1 to 34.7)	32.1 (26.2 to 38.5)	32.8 (28.0 to 38.1)
		East	31.9 (25.0 to 39.7)	39.7 (33.2 to 46.5)	38.8 (35.2 to 42.6)	35.0 (30.6 to 39.7)	39.9 (36.1 to 43.9)	46.4 (42.6 to 50.2)
		Central	30.3 (23.0 to 38.7)	26.6 (21.2 to 32.8)	40.2 (35.6 to 44.9)	38.1 (32.8 to 43.7)	31.3 (27.2 to 35.6)	42.5 (37.9 to 47.2)
		South	34.7 (28.8 to 41.1)	42.2 (30.9 to 54.3)	33.7 (26.0 to 42.4)	35.3 (27.2 to 44.5)	25.8 (19.5 to 33.3)	28.6 (24.2 to 33.5)
		Southwest	18.5 (13.6 to 24.8)	25.0 (21.8 to 28.4)	31.5 (26.2 to 37.3)	27.5 (23.7 to 31.6)	27.8 (23.7 to 32.5)	35.4 (31.3 to 39.8)
		Northwest	25.5 (19.6 to 32.6)	27.1 (20.6 to 34.7)	33.8 (24.0 to 45.4)	38.9 (33.8 to 44.3)	33.5 (29.1 to 38.2)	37.8 (31.0 to 45.1)
	Treatment	North	28.2 (21.8 to 35.7)	35.1 (28.5 to 42.3)	33.3 (28.6 to 38.5)	35.3 (31.4 to 39.4)	35.0 (31.8 to 38.4)	41.1 (32.3 to 50.4)
		Northeast	21.8 (18.0 to 26.2)	21.8 (17.1 to 27.4)	27.0 (22.2 to 32.4)	26.5 (22.7 to 30.8)	26.1 (21.7 to 31.0)	28.6 (23.9 to 33.9)
		East	26.5 (20.2 to 33.9)	31.7 (26.1 to 37.9)	30.6 (27.2 to 34.2)	31.5 (27.0 to 36.4)	34.3 (30.6 to 38.1)	41.6 (37.9 to 45.4)
		Central	22.8 (16.9 to 30.1)	21.2 (16.8 to 26.3)	30.4 (26.9 to 34.2)	33.1 (27.9 to 38.8)	27.1 (23.2 to 31.4)	39.1 (34.2 to 44.2)
		South	25.9 (20.9 to 31.5)	34.2 (22.8 to 47.9)	25.7 (18.6 to 34.3)	31.6 (23.8 to 40.5)	21.5 (14.9 to 29.9)	24.9 (21.1 to 29.3)
		Southwest	15.0 (10.9 to 20.2)	16.1 (12.5 to 20.5)	23.8 (19.1 to 29.3)	22.7 (18.9 to 26.9)	22.4 (19.0 to 26.3)	29.7 (25.6 to 34.1)
		Northwest	20.0 (14.3 to 27.2)	20.9 (15.4 to 27.8)	28.9 (20.2 to 39.5)	35.5 (31.0 to 40.3)	28.9 (25.0 to 33.1)	33.2 (26.1 to 41.2)
	Control	North	5.5 (4.3 to 7.1)	9.1 (5.1 to 15.8)	6.7 (4.9 to 9.2)	11.0 (8.4 to 14.2)	9.5 (7.8 to 11.5)	13.0 (9.5 to 17.6)
		Northeast	4.4 (3.1 to 6.2)	3.6 (2.7 to 4.9)	4.1 (3.3 to 5.1)	5.0 (3.4 to 7.3)	5.0 (3.5 to 6.9)	5.8 (4.1 to 8.2)
		East	9.2 (6.1 to 13.6)	9.1 (6.2 to 13.2)	5.9 (4.4 to 7.9)	11.8 (9.3 to 14.9)	11.4 (9.3 to 13.7)	15.4 (12.6 to 18.6)
		Central	5.3 (3.8 to 7.4)	5.9 (3.9 to 8.7)	6.5 (4.4 to 9.5)	10.3 (7.9 to 13.4)	5.7 (4.2 to 7.7)	13.1 (10.1 to 16.7)
		South	8.4 (5.8 to 12.0)	10.0 (4.7 to 19.9)	4.9 (3.9 to 6.2)	8.6 (6.3 to 11.6)	7.6 (4.6 to 12.4)	7.5 (5.6 to 9.9)
		Southwest	3.3 (1.7 to 6.3)	4.0 (2.4 to 6.6)	4.0 (2.9 to 5.7)	5.3 (3.6 to 7.6)	6.7 (4.6 to 9.7)	10.1 (7.6 to 13.3)
		Northwest	5.4 (3.3 to 8.7)	2.8 (1.5 to 5.3)	7.0 (3.9 to 12.4)	11.5 (9.4 to 13.9)	11.2 (7.7 to 16.0)	11.9 (8.5 to 16.5)
Women	Prevalence	North	31.4 (26.1 to 37.2)	30.9 (26.8 to 35.4)	44.7 (40.0 to 49.4)	36.2 (33.4 to 39.0)	34.5 (31.2 to 37.9)	30.0 (25.0 to 35.5)

Sex	Rate	Region	2004	2007	2010	2013	2015	2018
		Northeast	31.6 (26.2 to 37.6)	33.4 (28.5 to 38.8)	40.6 (36.2 to 45.1)	32.4 (27.4 to 37.7)	27.4 (23.4 to 31.9)	27.9 (21.2 to 35.8)
		East	22.1 (19.8 to 24.5)	29.2 (25.8 to 32.8)	35.4 (32.0 to 39.0)	26.2 (24.2 to 28.2)	28.8 (26.4 to 31.4)	23.2 (20.5 to 26.1)
		Central	22.9 (19.4 to 26.8)	34.4 (29.3 to 40.0)	32.8 (28.6 to 37.2)	29.1 (25.2 to 33.4)	28.9 (25.8 to 32.2)	27.1 (24.0 to 30.4)
		South	16.0 (12.4 to 20.5)	23.9 (20.7 to 27.4)	23.4 (21.1 to 25.9)	24.1 (20.5 to 28.2)	19.7 (16.1 to 23.9)	19.4 (16.9 to 22.1)
		Southwest	22.8 (19.1 to 27.1)	33.1 (27.5 to 39.2)	29.7 (24.1 to 36.1)	27.9 (24.9 to 31.0)	25.4 (23.6 to 27.2)	24.2 (22.1 to 26.5)
		Northwest	30.5 (26.5 to 34.8)	30.7 (26.9 to 34.7)	35.3 (31.5 to 39.3)	27.3 (24.3 to 30.5)	29.6 (26.4 to 33.0)	24.4 (21.8 to 27.3)
	Awareness	North	42.8 (34.4 to 51.7)	49.0 (42.6 to 55.5)	45.1 (39.1 to 51.3)	42.4 (38.6 to 46.2)	42.0 (37.3 to 46.8)	53.3 (47.4 to 59.1)
		Northeast	35.4 (31.1 to 39.9)	35.0 (28.7 to 41.9)	41.8 (35.9 to 47.9)	38.9 (33.5 to 44.7)	38.8 (35.6 to 42.1)	41.4 (37.0 to 46.0)
		East	40.9 (32.4 to 49.9)	43.0 (38.8 to 47.3)	40.9 (35.3 to 46.9)	38.1 (33.7 to 42.8)	43.5 (39.3 to 47.9)	50.7 (46.7 to 54.7)
		Central	35.7 (28.9 to 43.1)	30.9 (27.1 to 34.9)	43.5 (39.3 to 47.8)	38.8 (34.3 to 43.5)	34.9 (30.9 to 39.2)	45.3 (41.4 to 49.2)
		South	44.5 (39.4 to 49.7)	41.4 (34.2 to 49.0)	39.4 (30.0 to 49.6)	43.0 (35.6 to 50.8)	37.2 (24.9 to 51.5)	38.7 (33.1 to 44.6)
		Southwest	30.9 (25.0 to 37.5)	27.4 (21.8 to 33.7)	33.4 (28.2 to 39.0)	27.1 (23.3 to 31.2)	32.5 (28.6 to 36.8)	41.3 (36.4 to 46.5)
		Northwest	40.1 (26.9 to 54.9)	36.9 (30.3 to 44.0)	37.7 (30.6 to 45.3)	43.6 (38.5 to 48.9)	39.2 (33.4 to 45.4)	46.1 (41.1 to 51.2)
	Treatment	North	37.9 (31.2 to 45.1)	42.5 (36.4 to 48.9)	40.8 (35.2 to 46.8)	40.4 (36.7 to 44.3)	38.8 (34.4 to 43.3)	51.2 (45.1 to 57.2)
		Northeast	32.7 (28.7 to 37.1)	30.3 (24.5 to 36.9)	35.9 (31.3 to 40.7)	36.4 (31.5 to 41.6)	36.3 (33.8 to 38.9)	38.8 (34.5 to 43.3)
		East	36.6 (28.7 to 45.2)	35.1 (30.9 to 39.5)	36.5 (31.3 to 41.9)	35.7 (31.4 to 40.1)	40.1 (35.9 to 44.4)	47.2 (43.0 to 51.5)
		Central	32.3 (26.6 to 38.6)	24.0 (19.9 to 28.8)	38.6 (34.8 to 42.6)	36.7 (32.5 to 41.2)	31.5 (27.6 to 35.7)	42.6 (38.4 to 46.9)
		South	35.5 (27.1 to 45.0)	37.9 (30.3 to 46.1)	33.0 (23.5 to 44.0)	38.9 (31.6 to 46.6)	33.5 (20.7 to 49.2)	32.9 (29.0 to 37.2)
		Southwest	28.1 (22.3 to 34.8)	23.2 (19.6 to 27.2)	27.5 (22.9 to 32.7)	23.8 (20.1 to 27.9)	27.4 (24.3 to 30.8)	36.8 (31.3 to 42.7)
		Northwest	31.8 (22.1 to 43.3)	33.2 (26.4 to 40.9)	32.9 (26.1 to 40.5)	39.6 (35.3 to 44.1)	34.2 (28.7 to 40.1)	42.9 (37.6 to 48.5)
	Control	North	10.5 (7.9 to 13.8)	10.8 (7.0 to 16.3)	9.0 (6.3 to 12.7)	12.2 (9.6 to 15.3)	12.2 (10.2 to 14.6)	23.0 (15.7 to 32.4)
		Northeast	5.8 (4.3 to 7.9)	7.1 (5.0 to 9.9)	4.1 (2.6 to 6.6)	7.6 (5.3 to 10.7)	10.6 (8.9 to 12.7)	9.2 (6.2 to 13.5)
		East	10.1 (6.8 to 14.7)	7.7 (5.9 to 10.0)	7.8 (5.7 to 10.6)	12.8 (10.2 to 16.0)	14.2 (11.4 to 17.6)	17.8 (14.7 to 21.4)
		Central	6.2 (3.8 to 10.0)	5.9 (3.3 to 10.5)	6.9 (5.3 to 8.9)	9.6 (7.2 to 12.8)	7.7 (5.5 to 10.8)	12.0 (9.8 to 14.6)
		South	11.3 (8.7 to 14.5)	16.4 (8.7 to 28.8)	5.2 (2.4 to 10.6)	10.3 (7.5 to 13.9)	13.2 (6.7 to 24.2)	10.8 (7.8 to 14.7)
		Southwest	9.3 (5.9 to 14.5)	4.6 (3.6 to 5.8)	4.5 (2.8 to 7.2)	4.9 (3.5 to 7.0)	6.5 (4.8 to 8.8)	11.4 (7.4 to 17.2)
		Northwest	9.4 (5.6 to 15.1)	8.9 (5.3 to 14.5)	7.2 (4.2 to 11.9)	10.3 (8.0 to 13.1)	9.7 (6.2 to 14.6)	13.7 (9.8 to 18.9)

[a] The prevalence and rates were estimated using multivariable logistic regression containing age, survey year, sex, region, and 2-way and 3-way interaction terms of survey year, sex and region.

Supplementary Table 6. Trends in hypertension prevalence, awareness, treatment, and control rates by BMI groups (%)[a]

Sex	Prevalence	BMI (kg/m^2)	2004	2007	2010	2013	2015	2018
Men	Prevalence	<18.5	15.9 (12.5 to 20.1)	22.7 (14.9 to 33.0)	19.0 (15.7 to 22.7)	20.2 (12.1 to 31.8)	15.2 (12.2 to 18.7)	11.9 (9.0 to 15.7)
		18.5-	23.3 (21.2 to 25.5)	24.5 (22.5 to 26.6)	31.7 (29.8 to 33.7)	25.4 (24.0 to 26.9)	26.8 (25.0 to 28.6)	24.5 (22.9 to 26.2)
		25-	45.8 (42.0 to 49.5)	46.2 (43.1 to 49.4)	55.3 (53.3 to 57.2)	45.8 (43.9 to 47.8)	47.0 (44.6 to 49.4)	45.0 (43.0 to 47.0)
		30-	74.4 (68.3 to 79.6)	76.3 (70.7 to 81.1)	77.5 (74.8 to 80.0)	68.1 (65.1 to 70.9)	68.3 (64.2 to 72.0)	70.2 (66.4 to 73.8)
	Awareness	<18.5	13.6 (8.5 to 21.1)	12.2 (8.2 to 18.0)	14.9 (9.5 to 22.7)	18.7 (12.6 to 26.9)	21.0 (16.2 to 26.8)	23.8 (16.4 to 33.3)
		18.5-	22.6 (20.1 to 25.3)	28.9 (25.7 to 32.2)	29.9 (27.4 to 32.4)	29.1 (26.4 to 32.0)	27.6 (25.2 to 30.1)	33.7 (31.1 to 36.5)
		25-	40.2 (35.4 to 45.1)	41.4 (37.8 to 45.0)	43.2 (40.9 to 45.6)	39.0 (36.5 to 41.7)	38.7 (36.5 to 41.0)	43.5 (40.0 to 47.1)
		30-	50.9 (40.6 to 61.2)	51.7 (42.5 to 60.8)	48.6 (44.0 to 53.3)	43.9 (40.0 to 47.9)	44.1 (40.0 to 48.4)	50.4 (45.7 to 55.1)
	Treatment	<18.5	12.4 (7.4 to 20.0)	9.7 (6.0 to 15.3)	11.6 (6.7 to 19.5)	17.3 (11.4 to 25.3)	18.3 (13.8 to 23.9)	21.0 (13.8 to 30.8)
		18.5-	19.0 (16.7 to 21.6)	22.1 (19.0 to 25.7)	24.4 (22.3 to 26.6)	25.9 (23.3 to 28.8)	23.4 (21.2 to 25.7)	29.8 (27.3 to 32.5)
		25-	31.1 (26.4 to 36.2)	32.5 (28.7 to 36.5)	34.0 (31.7 to 36.4)	34.9 (32.4 to 37.4)	32.8 (30.4 to 35.3)	38.8 (35.0 to 42.8)
		30-	37.9 (27.7 to 49.2)	39.2 (32.4 to 46.4)	37.2 (33.0 to 41.6)	39.2 (35.4 to 43.1)	39.4 (35.1 to 44.0)	45.6 (41.7 to 49.5)
	Control	<18.5	3.5 (1.4 to 8.6)	2.3 (1.0 to 5.2)	3.3 (1.1 to 9.2)	4.9 (2.5 to 9.2)	4.5 (2.6 to 7.7)	6.9 (3.1 to 14.8)
		18.5-	6.0 (4.8 to 7.4)	6.0 (4.7 to 7.5)	5.6 (4.7 to 6.6)	8.7 (7.3 to 10.2)	8.4 (7.0 to 10.0)	11.1 (9.6 to 12.8)
		25-	6.9 (5.0 to 9.5)	8.3 (6.2 to 11.0)	6.2 (5.3 to 7.1)	10.7 (9.4 to 12.2)	8.8 (7.6 to 10.2)	12.9 (11.1 to 14.9)
		30-	8.4 (5.2 to 13.4)	8.8 (3.7 to 19.7)	4.5 (3.2 to 6.3)	9.6 (7.7 to 11.9)	8.4 (6.2 to 11.2)	10.9 (8.4 to 14.2)
Women	Prevalence	<18.5	9.3 (7.6 to 11.3)	15.3 (12.1 to 19.0)	14.4 (12.0 to 17.3)	10.8 (8.7 to 13.4)	12.2 (9.5 to 15.5)	8.3 (6.3 to 10.8)
		18.5-	20.2 (18.7 to 21.8)	24.2 (21.9 to 26.6)	25.7 (23.9 to 27.5)	21.5 (20.2 to 22.9)	20.3 (19.1 to 21.6)	17.6 (16.1 to 19.2)
		25-	35.0 (32.5 to 37.5)	45.1 (42.4 to 47.8)	48.6 (46.3 to 50.8)	37.4 (36.0 to 38.9)	37.1 (34.9 to 39.2)	34.4 (32.1 to 36.7)
		30-	56.9 (51.2 to 62.5)	60.9 (54.8 to 66.7)	67.1 (64.2 to 69.8)	54.0 (51.0 to 57.1)	54.5 (51.4 to 57.5)	46.4 (41.6 to 51.3)
	Awareness	<18.5	18.4 (11.9 to 27.5)	17.4 (9.6 to 29.4)	21.3 (15.0 to 29.3)	23.9 (17.0 to 32.5)	15.2 (10.8 to 21.1)	24.9 (18.4 to 32.7)
		18.5-	32.5 (28.3 to 37.0)	31.0 (26.6 to 35.8)	35.8 (32.8 to 38.9)	34.9 (32.4 to 37.6)	34.0 (31.6 to 36.5)	41.2 (38.7 to 43.9)
		25-	46.1 (41.9 to 50.3)	43.0 (39.5 to 46.6)	43.7 (41.2 to 46.3)	40.2 (37.9 to 42.4)	41.7 (39.3 to 44.1)	50.2 (47.3 to 53.1)
		30-	52.1 (45.4 to 58.7)	48.6 (42.4 to 54.8)	52.7 (49.2 to 56.3)	45.3 (41.8 to 48.9)	47.6 (43.6 to 51.7)	51.7 (48.1 to 55.2)
	Treatment	<18.5	12.9 (7.5 to 21.4)	10.7 (5.6 to 19.3)	17.6 (12.3 to 24.4)	20.5 (14.5 to 28.2)	10.0 (6.6 to 15.0)	23.6 (17.2 to 31.4)

Sex	Prevalence	BMI (kg/m^2)	2004	2007	2010	2013	2015	2018
		18.5-	28.4 (24.4 to 32.8)	25.8 (22.3 to 29.7)	30.7 (28.0 to 33.5)	32.2 (29.7 to 34.7)	30.7 (28.4 to 33.1)	38.1 (35.5 to 40.8)
		25-	40.6 (37.0 to 44.4)	36.5 (33.4 to 39.9)	38.9 (36.5 to 41.4)	37.6 (35.5 to 39.8)	37.8 (35.6 to 40.1)	46.8 (43.9 to 49.8)
		30-	46.9 (41.3 to 52.6)	41.9 (35.8 to 48.2)	47.0 (43.7 to 50.2)	42.4 (38.9 to 45.9)	43.2 (39.2 to 47.2)	48.6 (45.2 to 51.9)
	Control	<18.5	4.5 (2.0 to 9.6)	4.1 (1.5 to 10.6)	2.8 (1.4 to 5.3)	10.7 (6.1 to 17.9)	5.0 (2.5 to 9.6)	13.1 (7.9 to 21.0)
		18.5-	8.6 (6.8 to 10.8)	7.9 (5.5 to 11.2)	6.8 (5.6 to 8.2)	10.3 (8.8 to 11.9)	10.9 (9.3 to 12.7)	14.2 (12.2 to 16.6)
		25-	10.6 (8.6 to 13.1)	8.0 (6.5 to 9.7)	6.9 (5.7 to 8.2)	10.4 (9.1 to 12.0)	11.5 (10.0 to 13.2)	15.8 (12.6 to 19.6)
		30-	7.3 (5.5 to 9.7)	7.1 (4.5 to 11.1)	7.0 (5.3 to 9.2)	9.3 (7.9 to 11.0)	9.0 (5.8 to 13.8)	14.6 (12.4 to 17.1)

[a] The prevalence and rates were estimated using multivariable logistic regression containing age, survey year, sex, BMI, and 2-way and 3-way interaction terms of survey year, sex and BMI.

Supplementary Table 7. Trends in hypertension prevalence, awareness, treatment, and control rates by central obesity status (%)[a]

Sex	Prevalence	Central obesity [b]	2004	2007	2010	2013	2015	2018
Men	Prevalence	No	23.4 (21.1 to 25.8)	25.6 (23.7 to 27.6)	33.8 (31.8 to 35.8)	27.0 (25.7 to 28.3)	28.6 (26.8 to 30.6)	26.5 (24.7 to 28.4)
		Yes	50.8 (46.9 to 54.8)	50.2 (46.7 to 53.7)	61.5 (59.3 to 63.7)	50.8 (48.8 to 52.9)	52.5 (49.8 to 55.2)	51.7 (49.0 to 54.4)
	Awareness	No	22.6 (20.1 to 25.4)	28.5 (25.6 to 31.6)	31.7 (29.4 to 34.2)	29.7 (27.3 to 32.2)	29.4 (27.3 to 31.5)	34.8 (32.3 to 37.4)
		Yes	42.6 (38.4 to 46.9)	44.4 (40.4 to 48.4)	44.6 (42.2 to 47.1)	40.4 (37.9 to 42.9)	40.1 (37.6 to 42.6)	45.5 (42.1 to 48.8)
	Treatment	No	18.8 (16.4 to 21.3)	22.0 (19.0 to 25.3)	25.4 (23.4 to 27.6)	26.4 (24.1 to 28.9)	24.9 (23.1 to 26.9)	30.8 (28.3 to 33.5)
		Yes	33.0 (29.5 to 36.7)	34.4 (30.8 to 38.2)	35.1 (32.6 to 37.6)	36.1 (33.8 to 38.6)	34.4 (32.0 to 36.9)	40.8 (37.6 to 44.0)
	Control	No	5.8 (4.5 to 7.5)	5.8 (4.5 to 7.3)	5.7 (4.9 to 6.7)	8.6 (7.3 to 10.2)	8.3 (7.1 to 9.8)	10.8 (9.4 to 12.5)
		Yes	7.4 (6.0 to 9.1)	8.8 (6.3 to 12.2)	5.6 (4.5 to 6.8)	10.7 (9.5 to 12.1)	8.7 (7.6 to 10.0)	12.7 (11.0 to 14.7)
Women	Prevalence	No	19.6 (18.0 to 21.2)	25.5 (22.9 to 28.4)	27.0 (25.2 to 29.0)	21.4 (20.3 to 22.5)	20.8 (19.4 to 22.2)	17.7 (16.2 to 19.4)
		Yes	39.5 (36.4 to 42.7)	47.2 (44.7 to 49.7)	54.5 (52.3 to 56.6)	41.4 (39.5 to 43.3)	42.9 (41.2 to 44.6)	37.8 (35.6 to 40.0)
	Awareness	No	32.4 (28.6 to 36.5)	32.7 (28.8 to 36.8)	37.2 (34.6 to 39.8)	35.0 (32.4 to 37.6)	34.1 (31.9 to 36.3)	42.1 (39.6 to 44.6)
		Yes	47.2 (43.1 to 51.4)	43.4 (39.6 to 47.2)	45.4 (42.5 to 48.3)	41.2 (39.2 to 43.3)	43.6 (41.1 to 46.3)	50.0 (47.5 to 52.5)
	Treatment	No	28.3 (24.6 to 32.2)	27.1 (24.0 to 30.5)	31.9 (29.6 to 34.3)	32.3 (29.8 to 34.8)	30.4 (28.2 to 32.6)	38.9 (36.5 to 41.4)
		Yes	41.7 (38.5 to 44.9)	37.0 (33.5 to 40.6)	40.5 (37.8 to 43.3)	38.5 (36.5 to 40.5)	39.8 (37.2 to 42.5)	46.8 (44.1 to 49.5)
	Control	No	8.7 (6.8 to 10.9)	8.1 (6.0 to 10.8)	6.7 (5.6 to 7.9)	10.3 (8.9 to 12.0)	10.4 (8.9 to 12.1)	13.9 (11.9 to 16.1)
		Yes	9.6 (8.0 to 11.4)	7.3 (6.1 to 8.7)	6.9 (5.7 to 8.3)	10.2 (9.0 to 11.4)	11.3 (9.4 to 13.4)	15.8 (12.9 to 19.2)

[a] The prevalence and rates were estimated using multivariable logistic regression containing age, survey year, sex, central obesity, and 2-way and 3-way interaction terms of survey year, sex and central obesity.

[b] Central obesity was defined as having waist circumference ≥90 cm for men and ≥85 cm for women.

Supplementary Table 8. Trends in control rates among people under treatment for hypertension (%)

	Subgroups	2004	2007	2010	2013	2015	2018
Overall		26.6 (24.1 to 29.3)	25.6 (22.2 to 29.2)	19.2 (17.3 to 21.2)	29.6 (27.5 to 31.7)	30.2 (28.1 to 32.4)	33.7 (31.1 to 36.5)
Men	Overall	26.9 (23.0 to 31.3)	26.2 (22.2 to 30.7)	19.4 (17.4 to 21.6)	30.8 (28.3 to 33.4)	29.4 (26.9 to 32.2)	32.8 (30.2 to 35.6)
	Residence[a]						
	Urban	26.7 (21.4 to 32.7)	28.0 (22.6 to 34.2)	22.4 (19.7 to 25.5)	36.2 (32.3 to 40.3)	32.3 (28.6 to 36.2)	35.3 (31.5 to 39.4)
	Rural	27.3 (22.2 to 33.1)	22.8 (18.3 to 28.1)	15.1 (12.6 to 18.0)	26.4 (23.5 to 29.4)	25.0 (22.0 to 28.3)	29.0 (25.1 to 33.1)
	Education[a]						
	No Education	25.0 (14.2 to 40.2)	19.9 (14.2 to 27.0)	16.4 (13.2 to 20.2)	27.8 (23.0 to 33.3)	27.2 (22.8 to 32.1)	25.8 (21.4 to 30.7)
	Primary school	30.9 (20.7 to 43.3)	23.1 (16.2 to 31.9)	17.6 (14.1 to 21.7)	26.7 (22.9 to 30.9)	28.9 (24.6 to 33.6)	30.0 (25.8 to 34.6)
	Secondary school	25.0 (19.5 to 31.6)	26.3 (19.3 to 34.7)	18.2 (15.3 to 21.4)	29.8 (26.6 to 33.2)	28.0 (24.8 to 31.5)	33.5 (30.1 to 37.1)
	High school or above	27.1 (21.5 to 33.6)	31.2 (24.7 to 38.6)	23.8 (20.4 to 27.6)	37.8 (33.3 to 42.5)	33.6 (29.1 to 38.4)	37.1 (31.3 to 43.2)
	Age[b]						
	18-29	16.4 (0.0 to 38.7)	73.6 (41.0 to 100.0)	30.6 (17.6 to 43.5)	33.2 (15.5 to 51.0)	45.2 (21.2 to 69.1)	25.9 (3.8 to 47.9)
	30-39	50.7 (34.2 to 67.2)	33.2 (19.4 to 47.1)	21.4 (16.3 to 26.5)	29.1 (21.5 to 36.7)	35.2 (25.7 to 44.8)	45.2 (32.9 to 57.5)
	40-49	30.6 (23.6 to 37.7)	25.8 (20.3 to 31.3)	20.8 (18.1 to 23.6)	33.2 (29.5 to 36.9)	28.0 (23.2 to 32.8)	34.9 (29.2 to 40.6)
	50-59	25.9 (19.9 to 32.0)	27.0 (21.6 to 32.3)	21.5 (18.7 to 24.3)	33.5 (30.4 to 36.6)	32.6 (29.4 to 35.8)	31.3 (28.6 to 34.1)
	60-69	25.0 (19.8 to 30.2)	24.6 (19.4 to 29.8)	17.0 (14.9 to 19.2)	30.1 (27.0 to 33.2)	27.7 (25.1 to 30.2)	34.3 (31.9 to 36.6)
	Region[a]						
	North	19.4 (16.0 to 23.3)	25.8 (14.2 to 42.1)	20.0 (15.7 to 25.1)	30.7 (24.3 to 37.9)	27.1 (22.8 to 31.8)	31.4 (26.2 to 37.0)
	Northeast	20.3 (15.1 to 26.7)	16.9 (14.1 to 20.3)	14.7 (12.8 to 16.9)	18.5 (13.1 to 25.4)	18.9 (14.2 to 24.7)	20.1 (15.9 to 25.2)
	East	35.1 (27.7 to 43.2)	28.9 (22.7 to 36.0)	19.5 (15.7 to 24.1)	37.6 (32.9 to 42.4)	33.7 (29.6 to 38.1)	37.5 (32.5 to 42.7)
	Central	23.4 (18.2 to 29.5)	28.3 (20.1 to 38.2)	21.3 (15.5 to 28.6)	30.8 (26.2 to 35.8)	21.3 (16.6 to 26.8)	33.4 (28.4 to 38.8)
	South	32.2 (22.6 to 43.7)	29.4 (19.3 to 42.1)	19.2 (14.9 to 24.5)	26.8 (21.6 to 32.7)	35.8 (28.6 to 43.8)	30.7 (25.0 to 37.0)
	Southwest	21.4 (12.0 to 35.4)	24.8 (16.6 to 35.4)	16.8 (12.5 to 22.3)	22.8 (16.8 to 30.1)	29.7 (22.6 to 38.0)	34.0 (28.1 to 40.5)
	Northwest	27.2 (15.3 to 43.6)	13.7 (7.5 to 23.8)	24.0 (16.7 to 33.2)	31.7 (27.2 to 36.6)	39.2 (29.8 to 49.5)	36.3 (31.5 to 41.5)
	BMI (kg/m^2)[a]						
	<18.5	28.1 (11.2 to 54.7)	24.6 (10.5 to 47.4)	28.5 (12.4 to 52.7)	27.9 (14.6 to 46.6)	23.9 (13.9 to 37.8)	32.9 (15.5 to 56.7)
	18.5-	31.6 (26.7 to 36.9)	27.0 (22.4 to 32.0)	22.7 (19.9 to 25.8)	32.9 (28.8 to 37.3)	35.9 (32.0 to 40.1)	37.4 (33.5 to 41.5)
	25-	22.3 (16.9 to 28.9)	25.7 (20.1 to 32.2)	18.1 (15.8 to 20.7)	30.6 (27.9 to 33.5)	27.0 (24.0 to 30.2)	33.4 (30.7 to 36.3)
	30-	22.2 (13.6 to 34.1)	22.9 (10.2 to 43.6)	11.6 (8.3 to 15.9)	24.0 (19.7 to 28.8)	21.2 (15.9 to 27.7)	23.4 (17.7 to 30.1)
	Central obesity[a, c]						

	Subgroups	2004	2007	2010	2013	2015	2018
	No	31.0 (25.8 to 36.7)	26.4 (22.1 to 31.2)	22.7 (20.0 to 25.5)	32.2 (28.3 to 36.3)	33.6 (29.8 to 37.6)	35.5 (31.9 to 39.3)
	Yes	22.5 (18.2 to 27.5)	25.9 (19.7 to 33.2)	15.8 (13.2 to 18.8)	29.6 (27.0 to 32.3)	25.6 (22.8 to 28.5)	31.3 (28.2 to 34.5)
Women	Overall	26.3 (23.5 to 29.3)	24.9 (21.0 to 29.4)	18.9 (16.8 to 21.3)	28.4 (26.2 to 30.6)	31.0 (28.7 to 33.4)	34.6 (30.5 to 39.1)
	Residence[a]						
	Urban	28.5 (25.2 to 32.0)	28.6 (22.7 to 35.2)	24.0 (20.9 to 27.5)	35.8 (32.4 to 39.5)	36.8 (33.7 to 40.0)	41.7 (34.3 to 49.5)
	Rural	23.1 (18.9 to 27.9)	19.8 (16.2 to 24.0)	12.9 (11.1 to 15.1)	23.1 (20.8 to 25.6)	23.6 (21.0 to 26.5)	26.8 (23.9 to 29.8)
	Education[a]						
	No Education	19.4 (15.8 to 23.5)	17.8 (14.2 to 22.0)	13.9 (11.9 to 16.2)	24.9 (22.4 to 27.4)	25.4 (22.3 to 28.8)	28.3 (25.9 to 30.8)
	Primary school	26.3 (20.9 to 32.6)	26.0 (20.5 to 32.2)	15.7 (13.1 to 18.7)	26.6 (23.5 to 30.1)	30.9 (25.0 to 37.4)	30.9 (27.2 to 34.8)
	Secondary school	31.2 (24.6 to 38.6)	25.7 (20.0 to 32.4)	21.4 (18.0 to 25.2)	29.9 (25.9 to 34.2)	31.2 (27.0 to 35.7)	36.8 (29.7 to 44.5)
	High school or above	33.1 (26.1 to 40.9)	37.9 (27.0 to 50.2)	28.7 (24.0 to 34.0)	36.9 (31.8 to 42.2)	43.4 (38.6 to 48.2)	46.9 (38.5 to 55.6)
	Age[b]						
	18-29	49.6 (13.0 to 86.2)	42.5 (19.6 to 65.5)	50.2 (37.5 to 62.8)	39.9 (22.5 to 57.2)	59.5 (30.2 to 88.8)	25.4 (0.0 to 53.7)
	30-39	40.9 (27.0 to 54.7)	38.2 (23.9 to 52.5)	22.0 (15.8 to 28.2)	24.4 (16.4 to 32.3)	31.2 (20.7 to 41.8)	44.5 (25.8 to 63.3)
	40-49	26.4 (20.8 to 32.0)	24.6 (18.2 to 31.0)	20.0 (16.9 to 23.1)	31.1 (27.7 to 34.5)	35.9 (30.6 to 41.1)	32.5 (26.5 to 38.4)
	50-59	26.8 (22.0 to 31.5)	23.0 (17.0 to 29.0)	18.7 (16.1 to 21.3)	30.9 (28.5 to 33.2)	32.6 (29.4 to 35.8)	36.7 (32.0 to 41.4)
	60-69	24.2 (20.3 to 28.0)	25.8 (20.4 to 31.3)	17.6 (15.2 to 20.1)	26.1 (23.8 to 28.5)	27.3 (24.7 to 29.9)	34.4 (31.8 to 37.0)
	Region[a]						
	North	27.7 (22.8 to 33.2)	25.2 (17.9 to 34.2)	21.9 (16.6 to 28.4)	29.8 (24.3 to 35.8)	31.5 (27.5 to 35.9)	45.0 (33.9 to 56.6)
	Northeast	17.4 (12.8 to 23.2)	23.2 (17.6 to 29.9)	11.3 (7.4 to 16.7)	20.5 (15.5 to 26.6)	29.7 (25.6 to 34.1)	23.6 (15.5 to 34.0)
	East	27.5 (22.0 to 33.8)	22.0 (17.8 to 26.9)	21.5 (17.2 to 26.6)	35.6 (31.3 to 40.2)	36.0 (31.4 to 40.7)	38.1 (33.6 to 42.9)
	Central	19.0 (12.8 to 27.2)	24.9 (15.3 to 37.7)	17.7 (14.4 to 21.6)	25.9 (21.3 to 31.2)	24.6 (19.1 to 31.1)	28.5 (24.5 to 32.8)
	South	32.0 (23.9 to 41.3)	43.6 (25.8 to 63.2)	15.6 (8.4 to 27.1)	25.5 (20.1 to 31.8)	39.0 (30.5 to 48.2)	32.6 (24.8 to 41.5)
	Southwest	32.4 (23.9 to 42.2)	19.6 (14.6 to 25.8)	16.3 (11.0 to 23.6)	20.6 (16.1 to 26.1)	23.7 (18.5 to 29.7)	31.2 (22.3 to 41.7)
	Northwest	29.3 (23.2 to 36.3)	27.0 (17.2 to 39.7)	22.0 (15.5 to 30.2)	25.2 (20.3 to 30.9)	28.3 (21.0 to 37.0)	31.9 (24.8 to 39.8)
	BMI (kg/m^2)[a]						
	<18.5	32.8 (16.2 to 55.1)	37.3 (17.4 to 62.6)	15.7 (7.9 to 28.8)	48.8 (33.8 to 64.0)	47.4 (29.3 to 66.2)	54.4 (38.4 to 69.6)
	18.5-	30.0 (25.8 to 34.5)	30.5 (22.6 to 39.6)	22.1 (19.3 to 25.3)	31.4 (28.1 to 35.0)	35.5 (31.3 to 39.9)	37.5 (32.9 to 42.2)
	25-	25.9 (21.6 to 30.9)	21.7 (18.5 to 25.3)	17.5 (15.0 to 20.2)	27.2 (24.4 to 30.2)	30.5 (27.5 to 33.7)	33.7 (28.0 to 40.0)
	30-	15.6 (12.0 to 20.1)	16.8 (11.0 to 24.8)	14.8 (11.4 to 19.0)	21.8 (18.6 to 25.3)	21.0 (14.3 to 29.6)	30.1 (26.3 to 34.3)

Subgroups	2004	2007	2010	2013	2015	2018
Central obesity[a, c]						
No	30.2 (25.5 to 35.4)	29.8 (23.5 to 36.9)	20.9 (18.4 to 23.6)	31.6 (28.3 to 35.1)	34.3 (30.8 to 37.9)	35.8 (31.5 to 40.3)
Yes	23.2 (20.1 to 26.6)	19.7 (16.9 to 22.7)	17.1 (14.4 to 20.1)	26.0 (23.7 to 28.6)	28.5 (25.1 to 32.1)	34.0 (29.0 to 39.3)

[a] The prevalence and rates were estimated using multivariable logistic regression containing age, survey year, sex, variable of interest, and 2-way and 3-way interaction terms.

[b] Results were weighted to the 2010 census population in China.

[c] Central obesity was defined as having waist circumference ≥90 cm for men and ≥85 cm for women.

Supplementary Table 9. Trends in standardised hypertension prevalence (130/80 mmHg or diagnosed) in overall and by subgroups (%)[a]

	2004	2007	2010	2013	2015	2018
Overall	44.7 (42.1 to 47.4)	52.8 (50.4 to 55.2)	55.4 (53.3 to 57.5)	47.4 (46.0 to 48.8)	48.4 (46.2 to 50.5)	48.7 (47.1 to 50.3)
Male	50.8 (47.7 to 53.8)	57.5 (55.1 to 60.0)	61.1 (59.0 to 63.3)	53.9 (52.4 to 55.3)	56.7 (54.5 to 58.9)	58.8 (57.1 to 60.4)
Female	38.5 (36.0 to 41.0)	48.0 (45.1 to 50.8)	49.4 (47.2 to 51.6)	40.7 (39.1 to 42.3)	39.7 (37.4 to 42.0)	38.2 (36.1 to 40.4)
Urban	44.2 (40.4 to 48.0)	52.8 (49.1 to 56.5)	54.4 (51.5 to 57.2)	45.3 (43.0 to 47.7)	46.2 (42.8 to 49.7)	47.5 (45.0 to 50.0)
Rural	45.4 (41.8 to 49.0)	52.9 (49.9 to 55.9)	56.5 (53.5 to 59.6)	48.6 (46.9 to 50.3)	50.9 (48.9 to 52.8)	50.1 (48.3 to 51.9)

[a] Results were weighted to the 2010 census population in China.

Supplementary Table 10. Proportion of adults with hypertension who had SBP ≥160 mmHg or DBP ≥100 mmHg but were not diagnosed or treated (%)[a]

		2004	2007	2010	2013	2015	2018
Overall	Undiagnosed	15.7 (14.4 to 17.0)	18.6 (16.8 to 20.4)	18.8 (17.7 to 19.9)	18.6 (17.6 to 19.6)	17.9 (16.9 to 18.8)	15.3 (14.2 to 16.4)
	Untreated	21.0 (19.8 to 22.2)	21.6 (19.8 to 23.5)	21.9 (20.8 to 23.0)	20.0 (19.0 to 21.0)	19.7 (18.7 to 20.7)	16.9 (15.8 to 18.0)
Men	Undiagnosed	15.9 (14.3 to 17.4)	17.6 (15.8 to 19.5)	19.1 (17.9 to 20.3)	18.3 (17.2 to 19.5)	18.7 (17.5 to 19.8)	16.6 (15.1 to 18.0)
	Untreated	21.9 (20.2 to 23.5)	20.5 (18.5 to 22.6)	22.8 (21.6 to 24.1)	19.9 (18.8 to 21.1)	20.8 (19.5 to 22.0)	18.4 (16.8 to 20.0)
Women	Undiagnosed	15.4 (13.8 to 17.1)	19.6 (17.1 to 22.1)	18.4 (17.2 to 19.7)	19.0 (17.7 to 20.3)	16.7 (15.8 to 17.7)	13.4 (12.1 to 14.8)
	Untreated	20.0 (18.3 to 21.6)	22.8 (20.5 to 25.0)	20.8 (19.5 to 22.0)	20.1 (18.8 to 21.3)	18.3 (17.2 to 19.3)	14.7 (13.3 to 16.0)

[a] Results were weighted to the 2010 census population in China.

Supplementary Table 11. Trends in SBP, DBP and raised BP prevalence by age group[a]

Sex	BP	Age	2004	2007	2010	2013	2015	2018
Men	SBP (mmHg)	Overall	123.8 (122.8 to 124.7)	128.4 (127.6 to 129.2)	131.2 (130.3 to 132.1)	129.0 (128.4 to 129.5)	129.7 (128.8 to 130.5)	130.1 (129.4 to 130.7)
		18-29	117.5 (116.4 to 118.6)	122.1 (121.1 to 123.0)	123.6 (122.8 to 124.3)	123.5 (122.8 to 124.3)	123.3 (122.4 to 124.1)	125.3 (124.5 to 126.1)
		30-39	119.9 (118.9 to 120.8)	125.6 (125.0 to 126.2)	127.9 (126.9 to 128.9)	126.1 (125.5 to 126.8)	126.3 (125.2 to 127.5)	127.1 (126.4 to 127.8)
		40-49	124.1 (123.0 to 125.1)	129.1 (128.2 to 129.9)	132.2 (131.2 to 133.2)	129.1 (128.6 to 129.7)	130.6 (129.5 to 131.7)	130.4 (129.6 to 131.1)
		50-59	130.1 (129.0 to 131.3)	134.4 (133.4 to 135.4)	138.0 (137.1 to 138.9)	134.4 (133.8 to 135.1)	136.2 (135.3 to 137.0)	135.8 (134.8 to 136.7)
		60-69	138.1 (136.7 to 139.5)	140.6 (139.4 to 141.8)	145.4 (144.4 to 146.4)	139.8 (139.0 to 140.5)	141.7 (140.9 to 142.4)	139.7 (138.7 to 140.7)
	DBP (mmHg)	Overall	78.5 (77.7 to 79.3)	79.9 (79.4 to 80.4)	81.2 (80.7 to 81.8)	78.4 (78.0 to 78.8)	79.5 (79.1 to 80.0)	79.7 (79.3 to 80.2)
		18-29	73.9 (72.9 to 75.0)	76.5 (75.8 to 77.2)	76.1 (75.4 to 76.7)	74.2 (73.6 to 74.8)	74.4 (73.7 to 75.0)	75.2 (74.5 to 75.9)
		30-39	77.5 (76.7 to 78.4)	79.2 (78.6 to 79.8)	80.8 (80.2 to 81.3)	78.0 (77.4 to 78.5)	79.2 (78.5 to 79.9)	79.6 (79.0 to 80.1)
		40-49	80.8 (80.0 to 81.7)	81.4 (80.8 to 82.1)	83.8 (83.2 to 84.4)	80.5 (80.1 to 80.9)	82.4 (81.7 to 83.0)	82.3 (81.8 to 82.8)
		50-59	82.0 (81.1 to 82.8)	82.7 (82.0 to 83.4)	84.8 (84.3 to 85.3)	81.7 (81.3 to 82.1)	83.3 (82.9 to 83.7)	83.3 (82.7 to 83.8)
		60-69	81.5 (80.6 to 82.4)	82.7 (82.0 to 83.4)	84.5 (84.0 to 84.9)	80.2 (79.8 to 80.7)	81.6 (81.1 to 82.0)	80.8 (80.2 to 81.3)
	Raised BP prevalence (%)	Overall	21.1 (19.1 to 23.1)	22.8 (20.9 to 24.6)	30.6 (28.8 to 32.4)	25.1 (24.1 to 26.2)	26.3 (24.5 to 28.0)	26.0 (24.2 to 27.8)
		18-29	7.1 (5.3 to 8.9)	9.1 (7.2 to 11.0)	13.0 (11.2 to 14.7)	12.1 (10.6 to 13.6)	10.4 (8.6 to 12.1)	13.0 (10.9 to 15.0)
		30-39	13.6 (11.4 to 15.8)	15.9 (14.1 to 17.8)	24.6 (22.2 to 26.9)	19.6 (18.0 to 21.1)	19.0 (16.7 to 21.3)	18.8 (17.0 to 20.7)
		40-49	23.9 (21.2 to 26.6)	25.0 (22.8 to 27.3)	34.3 (32.3 to 36.2)	27.0 (25.6 to 28.3)	30.4 (28.1 to 32.6)	28.8 (27.0 to 30.5)
		50-59	34.8 (32.2 to 37.4)	36.1 (33.4 to 38.9)	45.9 (44.0 to 47.9)	37.7 (36.2 to 39.2)	41.3 (39.5 to 43.0)	40.4 (38.4 to 42.4)
		60-69	46.1 (43.2 to 49.0)	47.6 (45.1 to 50.1)	57.8 (55.8 to 59.8)	46.5 (45.0 to 48.1)	51.0 (49.4 to 52.6)	46.8 (44.6 to 49.1)
Women	SBP (mmHg)	Overall	119.8 (118.8 to 120.8)	125.7 (124.5 to 126.9)	126.2 (125.0 to 127.4)	124.2 (123.4 to 124.9)	123.9 (122.6 to 125.1)	122.6 (121.5 to 123.7)
		18-29	109.5 (108.4 to 110.6)	115.0 (113.9 to 116.1)	114.4 (113.2 to 115.7)	115.1 (113.9 to 116.2)	112.7 (111.5 to 114.0)	113.2 (112.2 to 114.2)
		30-39	113.8 (112.8 to 114.8)	119.5 (118.7 to 120.2)	120.0 (118.8 to 121.1)	117.9 (117.2 to 118.7)	117.3 (116.2 to 118.4)	116.3 (115.2 to 117.5)
		40-49	121.5 (120.4 to 122.6)	128.3 (127.1 to 129.6)	128.3 (127.3 to 129.3)	125.4 (124.8 to 126.1)	126.3 (125.5 to 127.2)	124.6 (123.4 to 125.8)
		50-59	131.2 (129.9 to 132.4)	136.8 (135.4 to 138.2)	138.9 (137.5 to 140.3)	134.6 (133.9 to 135.4)	135.9 (134.8 to 137.0)	133.5 (132.5 to 134.4)
		60-69	138.6 (137.3 to 139.9)	144.3 (142.7 to 145.8)	146.3 (145.1 to 147.5)	141.4 (140.5 to 142.2)	143.4 (142.3 to 144.4)	139.4 (138.2 to 140.5)
	DBP (mmHg)	Overall	75.0 (74.3 to 75.7)	78.3 (77.7 to 78.9)	78.8 (78.3 to 79.4)	74.7 (74.2 to 75.1)	74.7 (74.2 to 75.2)	74.2 (73.6 to 74.9)
		18-29	69.2 (68.4 to 70.0)	73.6 (72.8 to 74.4)	73.4 (72.7 to 74.1)	70.8 (70.1 to 71.4)	69.9 (69.3 to 70.5)	70.2 (69.4 to 70.9)

Sex	BP	Age	2004	2007	2010	2013	2015	2018
		30-39	73.1 (72.4 to 73.8)	76.4 (75.9 to 76.9)	77.1 (76.5 to 77.6)	73.0 (72.5 to 73.6)	73.1 (72.5 to 73.7)	72.7 (72.0 to 73.5)
		40-49	77.4 (76.6 to 78.2)	80.6 (79.9 to 81.3)	81.1 (80.6 to 81.6)	76.6 (76.1 to 77.0)	77.0 (76.5 to 77.4)	76.2 (75.5 to 76.9)
		50-59	80.6 (79.8 to 81.4)	82.5 (81.8 to 83.2)	83.9 (83.3 to 84.4)	78.5 (78.1 to 78.9)	79.4 (78.9 to 79.9)	78.4 (77.9 to 78.9)
		60-69	80.2 (79.3 to 81.1)	82.8 (82.2 to 83.5)	83.9 (83.3 to 84.5)	77.7 (77.3 to 78.2)	78.6 (78.1 to 79.1)	77.3 (76.6 to 77.9)
	Raised BP prevalence (%)	Overall	17.5 (16.0 to 19.0)	22.3 (20.2 to 24.3)	25.1 (23.3 to 26.9)	20.5 (19.4 to 21.5)	19.5 (18.1 to 20.9)	17.3 (15.7 to 18.8)
		18-29	2.4 (1.5 to 3.2)	5.3 (4.0 to 6.7)	6.5 (5.2 to 7.8)	7.2 (5.6 to 8.7)	3.1 (2.4 to 3.8)	3.9 (2.8 to 5.0)
		30-39	7.3 (6.0 to 8.6)	10.4 (9.1 to 11.7)	13.8 (12.2 to 15.4)	10.3 (9.2 to 11.4)	8.3 (7.4 to 9.3)	7.6 (6.0 to 9.1)
		40-49	19.5 (17.5 to 21.4)	25.7 (23.1 to 28.2)	27.9 (26.1 to 29.8)	20.6 (19.5 to 21.7)	21.7 (20.2 to 23.2)	18.6 (17.0 to 20.1)
		50-59	35.2 (32.7 to 37.7)	41.6 (38.7 to 44.5)	46.3 (43.6 to 49.0)	36.9 (35.6 to 38.3)	38.4 (36.3 to 40.4)	33.8 (31.9 to 35.6)
		60-69	47.3 (44.9 to 49.6)	55.1 (51.7 to 58.4)	59.4 (57.4 to 61.4)	49.7 (48.1 to 51.2)	52.5 (50.4 to 54.7)	44.8 (42.7 to 46.8)

[a] Results were weighted to the 2010 census population in China.

Supplementary Table 12. Model fitting and comparisons of linear, quadratic, cubic, and cubic spline model

		Linear model	Quadratic model[a]		Cubic model[b]		Spline model[c]	
		Adjusted R^2	*Adjusted* R^2	P^d	*Adjusted* R^2	P^d	*Adjusted* R^2	P^e
Overall	Hypertension	-0.07	0.39	0.14	0.35	0.30	0.54	0.13
	Awareness	0.45	0.50	0.32	0.78	0.21	0.84	0.15
	Treatment	0.70	0.83	0.14	0.82	0.30	0.82	0.19
	Control	0.53	0.83	0.07	0.76	0.26	0.81	0.10
Men	Hypertension	0.26	0.36	0.29	0.13	0.59	0.38	0.28
	Awareness	0.53	0.37	0.89	0.91	0.10	0.96	0.05
	Treatment	0.83	0.81	0.50	0.86	0.40	0.83	0.49
	Control	0.62	0.72	0.21	0.59	0.55	0.70	0.27
Women	Hypertension	-0.24	0.55	0.07	0.79	0.08	0.91	0.04
	Awareness	0.24	0.70	0.08	0.67	0.22	0.70	0.11
	Treatment	0.44	0.82	0.05	0.74	0.23	0.76	0.11
	Control	0.46	0.94	0.01	0.92	0.07	0.94	0.04
Urban	Hypertension	-0.05	0.08	0.30	0.04	0.46	0.21	0.28
	Awareness	0.19	0.29	0.30	0.14	0.53	0.26	0.33
	Treatment	0.64	0.61	0.47	0.44	0.78	0.64	0.50
	Control	0.57	0.55	0.44	0.40	0.70	0.57	0.44
Rural	Hypertension	-0.14	0.57	0.07	0.52	0.21	0.66	0.08
	Awareness	0.58	0.50	0.57	0.62	0.45	0.58	0.53
	Treatment	0.63	0.64	0.37	0.59	0.56	0.63	0.41
	Control	0.41	0.83	0.05	0.75	0.22	0.79	0.09

[a] Ordinary linear regression containing year and quadratic term of year.

[b] Ordinary linear regression containing year, quadratic term of year, and cubic term of year.

[c] Generalised additive model containing year and penalised cubic regression spline term of year with 3 basis functions.

[d] *P* value of ANOVA F-test to compare the linear model with quadratic and cubic models, respectively.

[e] *P* value of Wald test for equality to zero of cubic spline terms in spline model.
